# Ideomotor representation, motor control, and body aesthetics in juvenile rhythmic gymnasts: between reciprocal support and differentiated trajectories within a personalized training program

**DOI:** 10.3389/fspor.2026.1874791

**Published:** 2026-07-20

**Authors:** R. M. Ciorășteanu, R. Mijaica, L. Balint

**Affiliations:** 1 Interdisciplinary Doctoral School, Transilvania University of Brașov, Brașov, Romania; 2 Department of Physical Education and Special Motricity, Faculty of Physical Education and Mountain Sports, Transilvania University of Brașov, Brașov, Romania

**Keywords:** body aesthetics, competitive performance, ideomotor representation, integrative training, juvenile rhythmic gymnastics, motor control

## Abstract

**Objective:**

This study aimed to evaluate the effects of a personalized integrative training program on ideomotor representation, motor control estimated through whole-routine performance, and body aesthetics in juvenile rhythmic gymnasts, as well as to examine short-term transfer to competitive performance.

**Methods:**

The study used a pre–post intervention design and included 9 competitive rhythmic gymnasts aged 8–11 years who followed, for 14 weeks, a program integrated into their regular training. The assessment included whole-routine performance, used as an operational expression of motor control, computerized video analysis, the Movement Imagery Questionnaire-3 (MIQ-3), and the Evaluation of Body Aesthetics in Sports (EBAS).

**Results:**

All athletes showed increases in total whole-routine score, with the group mean rising from 16.06 ± 1.10 to 17.26 ± 1.00 points, while the mean difficulty realization rate increased from 64.66% to 72.82%. At the inferential level, ideomotor representation was the only variable that showed a statistically significant change between T0 and T2 (Z = −2.25, *p* = 0.024). Indicators of whole-routine performance and body aesthetics showed favorable directional improvements, but these were not statistically significant at group level. At the individual level, the response to the intervention was differentiated, and short-term transfer to competitive performance remained variable.

**Conclusion:**

Ideomotor representation was the dimension most responsive to the intervention and may provide a useful reference point for optimizing performance organization in juvenile rhythmic gymnasts, even though the relationships among ideomotor representation, motor control, and body aesthetics remain unevenly consolidated in the short term and transfer to competitive performance is expressed differentially.

## 1 Introduction

In juvenile rhythmic gymnastics, performance results from the coordination of physical, technical, psychological, and artistic demands within an execution that is coherent, stable, and expressive. In this discipline, performance quality depends not only on the difficulty of the elements or on the level of physical preparation, but also on the athlete's ability to integrate postural control, the body–apparatus relationship, synchronization with music, expressiveness, and technical stability within the structure of the competitive routine (1–3). In this sense, performance reflects the integration of technical difficulty, execution quality, and artistic expression within a regulatory framework that explicitly combines these dimensions (1, 2, 4). Maintaining execution quality under demanding conditions depends on efficient motor control, supported by sensory integration, action anticipation, error correction, self-regulatory mechanisms, and the appropriate use of feedback, alongside the development of physical capacities (5–8).

Beyond motor control itself, performance in rhythmic gymnastics also involves an internal component of movement anticipation and organization, expressed through ideomotor representation.

The concept of ideomotor representation originates from ideomotor theories of action, which propose that movements are cognitively represented through their anticipated sensory consequences and action effects. Contemporary approaches in motor cognition describe ideomotor representation as an internal organization of action that links perception, anticipation, movement planning, and the mental simulation of future actions, allowing individuals to evaluate, regulate, and refine motor behavior before or during execution. In this sense, ideomotor representation can be understood as the internal simulation or mental rehearsal of movement in the absence of actual execution, with a role in the anticipation, organization, and regulation of action (9–12).

Research in the field of motor imagery shows that mental representations of movement activate functional mechanisms that are partly shared with those involved in actual execution and may facilitate the learning, consolidation, and refinement of performance, especially when they are integrated with physical practice and adapted to the specific features of the task (9, 10, 13). In children and preadolescents, this capacity is still developing and is influenced by age, motor experience, and level of cognitive maturation, which gives particular relevance to interventions applied in youth sport (14, 15).

From an applied perspective, one of the ways in which ideomotor representation can be stimulated and systematically integrated into the training process is mental practice. The literature shows that its effects tend to be more consistent when the imagined content maintains a high degree of functional correspondence with actual execution conditions and remains directly associated with the target motor task (16–18). Within this logic, the Physical, Environment, Task, Timing, Learning, Emotion, Perspective (PETTLEP) model proposes that mental practice should be structured so as to reproduce as faithfully as possible the physical, temporal, emotional, and contextual components of execution, which makes it particularly useful in disciplines in which precision and expressiveness must be supported simultaneously (17, 18). In addition, video observation may provide useful support both for execution analysis and for instruction, because it facilitates guided observation, model–execution comparison, error detection, and the consolidation of movement representation, especially when associated with mental practice and action observation (19–21). In beginner gymnastics learning contexts, the use of video feedback has been associated with improvements in motor learning, self-assessment, and motivation, which supports the relevance of this tool in instructional and self-regulatory processes (22). Overall, the available evidence suggests that combining mental practice with observation and with actual motor execution may foster the development of ideomotor representation and its transfer to performance (16, 19, 23).

In rhythmic gymnastics, these demands also extend to the artistic component, and body aesthetics represents a defining dimension of execution, expressed through posture, amplitude, fluidity, expressiveness, and the coherent relationship between movement and music (2, 24). In this context, the relationship between musical phrasing and movement organization contributes to the expressive coherence of the routine and to the articulation of artistic components during execution (25). From this perspective, body aesthetics is not simply a decorative addition to technique, but rather reflects the level of integration among motor control, movement organization, and expressive intention (26). Studies on artistic and choreographic preparation show that this dimension can be developed and optimized through specific means, including dance, expressive exercises, and the use of music as support for the structuring and internalization of movement (27–30).

Although the literature supports the role of each of these components, approaches often remain fragmented, treating physical and technical preparation, motor control, motor imagery, and artistic expressiveness separately. In training practice, such fragmentation may limit transfer to the whole routine, especially in young athletes, for whom demands related to coordination, regulation, expressiveness, and adaptation manifest simultaneously. Moreover, although several studies have separately highlighted the role of motor control, artistic preparation, or motor imagery, integrated interventions explicitly examining ideomotor representation in relation to whole-routine performance, video analysis as support for assessment and instruction, and body aesthetics in juvenile rhythmic gymnasts remain limited. This gap is important from both theoretical and applied perspectives, because it is precisely in this age category that the relationship among the internal organization of movement, execution control, and bodily expressiveness is still being consolidated.

In line with this perspective, previous research on rhythmic gymnasts has highlighted, from complementary angles, the relevance of physical and coordinative components in the early stages of training, the importance of postural control and static balance, and the role of body aesthetics in supporting artistic expression and execution quality (31–34). In addition, studies on the development of motor imagery and the organization of action representations indicate the need for more explicit investigation of this dimension in the context of juvenile rhythmic gymnastics (12, 15, 35). Taken together, these contributions support the need for integrated interventions explicitly designed to examine how ideomotor representation relates to whole-routine performance and body aesthetics in the actual unfolding of movement.

From an applied perspective, such an articulation cannot remain at the level of simple coexistence of components in training, but requires rational and explicit programming, materialized in planning documents and structured interventions aimed at the progressive coordination of these components within the structure of the competitive routine. In this context, interventions that systematically integrate mental practice with physical and technical preparation deserve investigation, particularly when ideomotor representation is placed at the center of execution organization and regulation.

The aim of the present study was to evaluate the effects of a personalized integrative training program on ideomotor representation, motor control estimated through whole-routine performance, and body aesthetics in competitive juvenile rhythmic gymnasts, as well as to examine short-term transfer to competitive performance. We hypothesized that the implementation of such a program would lead to improvements in ideomotor representation capacity and to favorable developments in the other components under investigation, even if the magnitude and pace of these changes might differ.

## 2 Materials and methods

### 2.1 Study design

The study used a pre–post intervention design conducted within an exploratory multiple-case framework, appropriate to the small sample size and the mixed composition of age categories. The study aimed to evaluate the effects of a personalized integrative training program on ideomotor representation, motor control estimated through whole-routine performance, and body aesthetics in athletes competing in individual rhythmic gymnastics events at a competitive level.

The protocol included two formal assessment moments: an initial assessment (T0) and a final post-intervention assessment (T2), separated by a 14-week intervention period; no intermediate T1 outcome assessment was conducted. During the intervention period, the athletes participated in a personalized integrative training program incorporated into their regular training process. The program combined motor-control exercises, body-aesthetics training, technical preparation, whole-routine execution, competition simulations, individualized feedback, and PETTLEP-based ideomotor training and motor imagery. A detailed description of the intervention structure is provided in Section 2.4. Official competition results were also collected to examine the ecological transfer of the intervention. Given the small sample size and the heterogeneity of the group, the interpretation of the findings had an exploratory character, combining group-level analysis with a succinct description of individual trajectories.

### 2.2 Participants and ethical considerations

The intervention and primary pre–post assessments were conducted between February and May 2023 at ACS Ritmic Aly Gym Club, affiliated with the Romanian Rhythmic Gymnastics Federation. Official competition results from the 2023 season were subsequently collected for the exploratory analysis of ecological transfer. The inclusion criteria were: valid sports registration, regular attendance at training sessions during the previous six months, health status compatible with physical exertion, availability to complete the pre–post assessment and intervention stages, and written informed consent provided by parents/legal guardians. Athletes with recent injuries or medical conditions incompatible with physical effort, those with irregular training attendance, those who did not complete the study stages, and those for whom the required approvals were not obtained were excluded. Out of a total of 24 registered and active club athletes, 9 participants met the inclusion criteria and completed the pre–post research protocol, representing 37.5% of all active athletes in the club. The mean age of the group was 9.56 ± 0.88 years, ranging from 8 to 11 years, and mean sport experience was 2.78 ± 1.09 years, ranging from 1 to 4 years.

The group included athletes from three competitive categories used in the Romanian national rhythmic gymnastics system: 1 gymnast in Junior IV, 7 gymnasts in Junior III, and 1 gymnast in Junior II. In the 2023 national competition framework, Junior IV corresponded to gymnasts aged 6–8 years, Junior III to gymnasts aged 9–10 years, and Junior II to gymnasts aged 11–12 years. These categories represent successive youth competitive levels within the national system, while the technical construction and judging of routines followed the applicable rhythmic gymnastics competition requirements. To ensure data traceability, each participant was assigned a unique code that was maintained throughout the study.

The study was approved by the Ethics Committee of the Faculty of Physical Education and Mountain Sports, Transilvania University of Brașov, through Decision No. 425/20.12.2022, and was conducted in accordance with the ethical principles of research involving human participants and with the provisions of the Declaration of Helsinki. Participation in the study took place with the agreement of the management of ACS Ritmic Aly Gym Club and with the informed consent of parents/legal guardians, as all participants were minors. Parents/legal guardians were informed about the purpose, methodology, and stages of the study, the voluntary nature of participation, and the fact that personal data would be kept confidential and used exclusively for scientific purposes. Parental consent covered participation in testing, questionnaire completion, video-based evaluations, and the training program integrated into the study. Participants received age-appropriate explanations regarding the purpose and procedures of the research, and participation took place with their assent. Personal identifiers were removed from the analytical dataset, and questionnaires, individual records, and participants' results were handled using the assigned codes and were collected, stored, and used exclusively for the purposes of the study under conditions of confidentiality.

### 2.3 Variables, instruments, and assessment procedures

Operationally, the independent variable was the personalized integrative training program applied between T0 and T2. The main dependent variables were: (a) ideomotor representation; (b) body aesthetics; and (c) motor control estimated through whole-routine performance. The secondary dependent variable was competitive performance, operationalized through the official score obtained in competition and/or ranking position. Computerized video analysis had a complementary role, supporting execution analysis, interpretation of individual progress, and technical-artistic feedback. The main assessments (MIQ-3, EBAS, whole-routine performance, and computerized video analysis) were conducted at T0 and T2 under standardized conditions, in the same hall and on the same working surface, with the same sequence of tests, the same warm-up conditions, and the same general administration and execution instructions. Competitive performance was analyzed separately, based on official competition results, according to the criteria described in Section 2.3.5.

#### 2.3.1 Ideomotor representation: MIQ-3

Ideomotor representation was assessed using the Movement Imagery Questionnaire-3 (MIQ-3), a psychometric instrument used to estimate the ease with which a participant can generate motor images in three modalities: internal visual imagery, external visual imagery, and kinesthetic imagery. In the present study, MIQ-3 scores were used as indicators of ideomotor representation capacity. The MIQ-3 is a general multidimensional measure of movement imagery ability and was not developed specifically for rhythmic gymnastics. Its original development and validation supported its three-factor structure and provided evidence of concurrent and predictive validity (35). Further support for the use of MIQ-3 in athletic populations is provided by Williams and Cumming (36), whose Study 4 administered the MIQ-3 together with the Sport Imagery Ability Questionnaire to 220 athletes from 30 different sports and reported good internal reliability for the MIQ-3 subscales, as well as significant bivariate correlations between SIAQ and MIQ-3 subscales.

The version described by Williams et al. (35) was used, translated into Romanian with minor wording adaptations for linguistic clarity. These adaptations were limited to linguistic clarification; the item content, response format, and scoring procedure were not modified, and no discipline-specific adaptation or validation for rhythmic gymnasts was undertaken.

The instrument comprises four simple movements, each imagined in three modalities, resulting in 12 items rated on a 7-point Likert scale. Higher scores indicate greater self-reported ease in generating motor images across the three modalities assessed. Scores were calculated separately for each dimension as the mean of the corresponding items.

MIQ-3 was administered individually at T0 and T2 under confidential conditions, without suggesting responses, and under standardized supervision. In the case of younger participants, instructions were delivered uniformly and exclusively to clarify the task, without altering the wording of the items or the meaning of the response options.

MIQ-3 showed very good internal reliability in the present sample, with Cronbach's alpha = 0.923, and all items were retained in the analysis.

#### 2.3.2 Body aesthetics: EBAS

Body aesthetics was assessed using the Evaluation of Body Aesthetics in Sports (EBAS), an instrument developed within the present research for the specific context of rhythmic gymnastics, drawing on the literature on body image, associated aesthetic dimensions, and the particularities of aesthetic sports (37–41). In the present study, EBAS scores were used as indicators of perceived body aesthetics.

The instrument includes 20 unidirectionally worded items rated on a 5-point Likert scale. The items were organized into five thematic sections: perception of body aesthetics; influence of training on body aesthetics; harmony of movement; ideomotor representation and self-control; and posture and body line. In the present analysis, EBAS was treated as a global scale, while the five sections had a descriptive role for further characterizing the response profile, without being used as independent psychometric subscales.

The global EBAS score was calculated as the mean of the 20 items. Higher scores indicate a more favorable perception of body aesthetics, namely a higher perceived level of harmony, expressiveness, and aesthetic quality of bodily execution. The instrument did not include reverse-worded items; therefore, no reverse coding was required.

EBAS was administered individually at T0 and T2 under the same administration conditions as MIQ-3, with respect for confidentiality and standardized instructions. EBAS showed very good internal reliability, with Cronbach's alpha = 0.861. For the estimation of internal consistency, 19 items were analyzed because Item 5 showed zero variance in the analyzed sample and did not contribute to differentiating participants. However, the item was retained in the descriptive scoring and in the calculation of the global EBAS score; its exclusion applied exclusively to the estimation of the alpha coefficient.

#### 2.3.3 Motor control and whole-routine performance

Motor control was estimated through performance in the whole rhythmic gymnastics routine, performed under competition-like conditions. In the present study, whole-routine performance was treated as an operational indicator of motor control in a competition-specific context.

Each participant was assessed using one whole routine with apparatus, selected and kept constant between T0 and T2 in order to standardize the procedure and ensure comparability of intra-individual progress. At each testing moment, each athlete performed two complete executions of the routine, and the execution with the higher total score was retained for analysis.

The routines were scored according to FIG judging logic, using the components of difficulty (D), artistry (A), and technical execution (E). In the main quantitative analysis, execution quality was expressed through the composite execution score, defined as A + E, while the total routine score was calculated as follows: Total score = D + A + E−penalties. Higher scores indicate better global execution, expressed through more effective realization of difficulty and higher quality of the artistic and technical components, within the judging criteria applied.

The evaluation was conducted at T0 and T2 by the same judging panel, consisting of two international judges holding Brevet IV valid for the 2022–2024 Olympic cycle and three judges holding National Brevet I, in order to limit procedural variation and ensure comparability between the two time points.

#### 2.3.4 Computerized video analysis

The assessment was complemented by computerized video analysis performed using myDartfish 360 software. Filming of the whole routines was standardized by maintaining the same camera position relative to the competition surface, so that the entire execution could be captured. Video materials were labeled uniformly using the participant code, assessment moment, and apparatus used.

Video analysis had a complementary role and was used both for the qualitative and quantitative examination of executions at T0 and T2 and to support individualized feedback during the intervention. In particular, movement amplitude, execution fluency, accuracy of technical elements, bodily expressiveness, and body–apparatus–music synchronization were monitored. Depending on the case, the analysis also included the measurement of relevant joint angles in balance, jump, and rotation elements. The indicators extracted through video analysis had a complementary role in the interpretation of individual progress, without constituting distinct main inferential variables.

#### 2.3.5 Competitive performance (ecological transfer)

Competitive performance was treated as a secondary dependent variable used to examine the ecological transfer of the intervention. It was operationalized through the official results obtained in the All-Around event at the main competition of the season, the National Championship, expressed as total score and ranking position. In addition, in order to capture the expression of progress outside the target competition, the best result obtained by each athlete in the secondary competitions of the 2023 season was also taken into account. The data were retrieved from the official competition rankings. Because participation in secondary competitions varied across athletes, due to selection criteria, age category, season management, and logistical aspects, the best available result was retained for each participant for comparability purposes. These indicators had a complementary and exploratory role and were used to describe competitive performance and to analyze its relationship with the Coefficient of Harmony (CA) and the Weighted Coefficient of Harmony (CA-P), synthetic indicators of the functional profile calculated subsequently on the basis of the central components assessed.

### 2.4 Personalized integrative training program and monitoring framework

The personalized integrative training program was incorporated into the annual training plan for the 2023 competitive season. Within the present study, the experimental monitoring covered a 14-week window extending from February to May, prior to the target competition. From a periodization perspective, this sequence corresponded to the basic and refinement mesocycles and included the beginning of the pre-competitive mesocycle.

Operationally, February corresponded to the initial assessment (T0) and the beginning of the personalized program, March to the consolidation and extension phase of the intervention, April to the intensified integration of the contents into routine segments and whole-routine executions, and the first part of May to pre-competitive fine-tuning and the final assessment (T2). Pre–post monitoring was restricted to this temporal sequence. The subsequent stages of the annual plan—continuation of the pre-competitive phase, the competitive period, and transition—followed the club's usual training logic, without a full repetition of the initial assessments and without recalculation of the synthetic indicators.

The program did not function as a separate imagery-training module, but was integrated into regular training. During the monitored interval, the intervention was implemented in four weekly sessions, with approximately 11 h of training per week, totaling 154 h. Of this volume, 33 h were allocated to motor control, 31 h to body aesthetics, and 14.5 h to ideomotor representation, while the remaining time was distributed among specific technical preparation, whole-routine executions, competition simulations, and individualized feedback and correction sequences. Overall, the three central components amounted to 78.5 h, representing 51.0% of the monitored volume and 26.37% of the annual planned training volume (584 h).

The intervention was individualized on the basis of each athlete's initial profile, constructed from the results obtained at T0 for the whole routine, EBAS, and MIQ-3. In the case of execution, particular consideration was given to realized difficulty in relation to scripted difficulty and to the composite execution score. On this basis, individual intervention priorities were established so that each participant received differentiated emphasis on the component or components considered limiting at baseline.

The content of the program was organized around three fundamental components: motor control, body aesthetics, and ideomotor representation. The motor control component targeted postural stability, balance, control of the bod*y* axis, precision in apparatus handling, and reduction of technical errors. The body aesthetics component targeted posture, body line, fluency of transitions, coordination with music, and expressiveness. The ideomotor representation component included structured motor imagery sequences applied to technical segments, artistic sequences, and the whole routine. The central logic of the intervention was based on a “mosaic” structure, in which these components were functionally combined within the same training session in order to support direct transfer to the whole routine.

The internal progression of the intervention followed a simple-to-complex sequence. In the first stage, the emphasis was placed on accumulation, standardization, and consolidation of the foundations of motor control and body aesthetics, with the gradual introduction of ideomotor representation sequences. In the middle stage, the emphasis shifted toward integrating the components into routine segments and increasing the proportion of combined sequences. In the final, pre-competitive stage, the focus was on stabilizing execution under competition-like conditions, increasing the proportion of whole-routine executions and simulations, fine adjustment of execution details, and management of emotional state.

The development of ideomotor representation was supported through structured motor imagery techniques, with integration of the principles of the PETTLEP model (17, 18). Application of this model was operationalized through the mental representation of the whole routine or of relevant sequences under conditions as close as possible to the actual execution context: the real starting position, apparatus in hand, the training environment, the routine music, the actual duration of the sequence, alternation between internal and external perspective, and inclusion of the competitive emotional component. These sequences were used mainly 2–3 times per week, especially before key segments, whole-routine executions, and simulations. In addition, video observation was used to support comparative analysis of executions, individualized feedback, and consolidation of movement representation during the instructional process.

Adherence to the intervention was monitored systematically through the attendance register and individual work sheets in order to support the correct interpretation of individual progress. In addition to the results obtained at the target competition, official results from the secondary competitions of the 2023 season were also collected and used exploratorily to examine the relationship between the indicators calculated at T2 and competitive performance.

### 2.5 Statistical analysis and synthetic indicators of multidimensional harmony

The data were analyzed by reporting descriptive indicators for all variables under investigation. The normality of the distributions was checked using the Shapiro–Wilk test, which is appropriate for small samples. Depending on data distribution, the Wilcoxon paired-samples test was used for variables that did not meet the normality assumption, whereas paired-samples t-tests were used for variables with distributions compatible with normality. Statistical analyses were performed using SPSS v.21, while Microsoft Excel 2010 was used for data centralization, auxiliary checks, and graphical representations. The level of statistical significance was set at *p* < 0.05.

Effect size was expressed as Cohen's d for parametric analyses and as r for the Wilcoxon analysis (54). To evaluate individual changes in MIQ-3 and EBAS, the Reliable Change Index (RCI) was calculated. In addition, for the global analysis of progress, two synthetic indicators developed within the research were used: the Coefficient of Harmony (CA) and the Weighted Coefficient of Harmony (CA-P). These indicators had a descriptive and exploratory role and were used to estimate the degree of reciprocal support among the three central dimensions of the functional profile: whole-routine performance, the EBAS score, and the MIQ-3 score.

In a first stage, the Coefficient of Harmony (CA) was calculated as a basic indicator of reciprocal support among the three components treated with equal weights. For each athlete, the raw values corresponding to Total score, EBAS, and MIQ-3 were transformed into standardized z scores using the parameters of the initial time point (*μ*_T0, *σ*_T0), so that the values at T0 and T2 were referred to the same reference base. Subsequently, the internal dispersion of the three standardized scores was calculated for each individual profile, and CA was obtained by normalizing this dispersion to the maximum empirical value of the dispersion established on the combined T0–T2 dataset. The formula used was:

$$CA=1-\frac{SD(z\_ST,\;z\_EBAS,\;z\_MIQ\_3)}{SDmax}$$

In this equation, ST denotes the total whole-routine score, SD represents the standard deviation of the three z scores of the same athlete, and SDmax is the maximum empirical value of the dispersion used to normalize the indicator to the [0,1] interval. Higher CA values indicate more internally balanced profiles, that is, stronger reciprocal support among the analyzed components.

In a second stage, the Weighted Coefficient of Harmony (CA-P) was calculated on the same logic, but in a version in which the three components were weighted differentially. Whole-routine score was introduced as an operational expression of competition-relevant motor control, EBAS for the aesthetic component, and MIQ-3 for the ideomotor component. The weights were established *a priori* as follows: whole-routine score = 0.26, EBAS = 0.33, and MIQ-3 = 0.41, in accordance with the analytical model of the research and with the functional relevance attributed to each dimension. After standardization, the scores were weighted (z′ = z   ×   p), and the internal dispersion of the weighted profile was calculated for each athlete. The formula used was:

$$CA\_P=1-\frac{SD\_p\;(z^{\prime }\_ST,\;z^{\prime }\_EBAS,\;z^{\prime }\_MIQ\_3)\;}{SDmax,p}$$

In this equation, SD_p represents the standard deviation of the three weighted scores of the same athlete, and SDmax,p represents the maximum empirical value of SD_p in the combined T0–T2 weighted profiles. Higher CA-P values indicate more internally balanced weighted profiles. The relationships between CA, CA-P, and competitive performance were examined using Spearman correlations.

## 3 Results

### 3.1 Ideomotor representation (MIQ-3)

**Figure 1:**
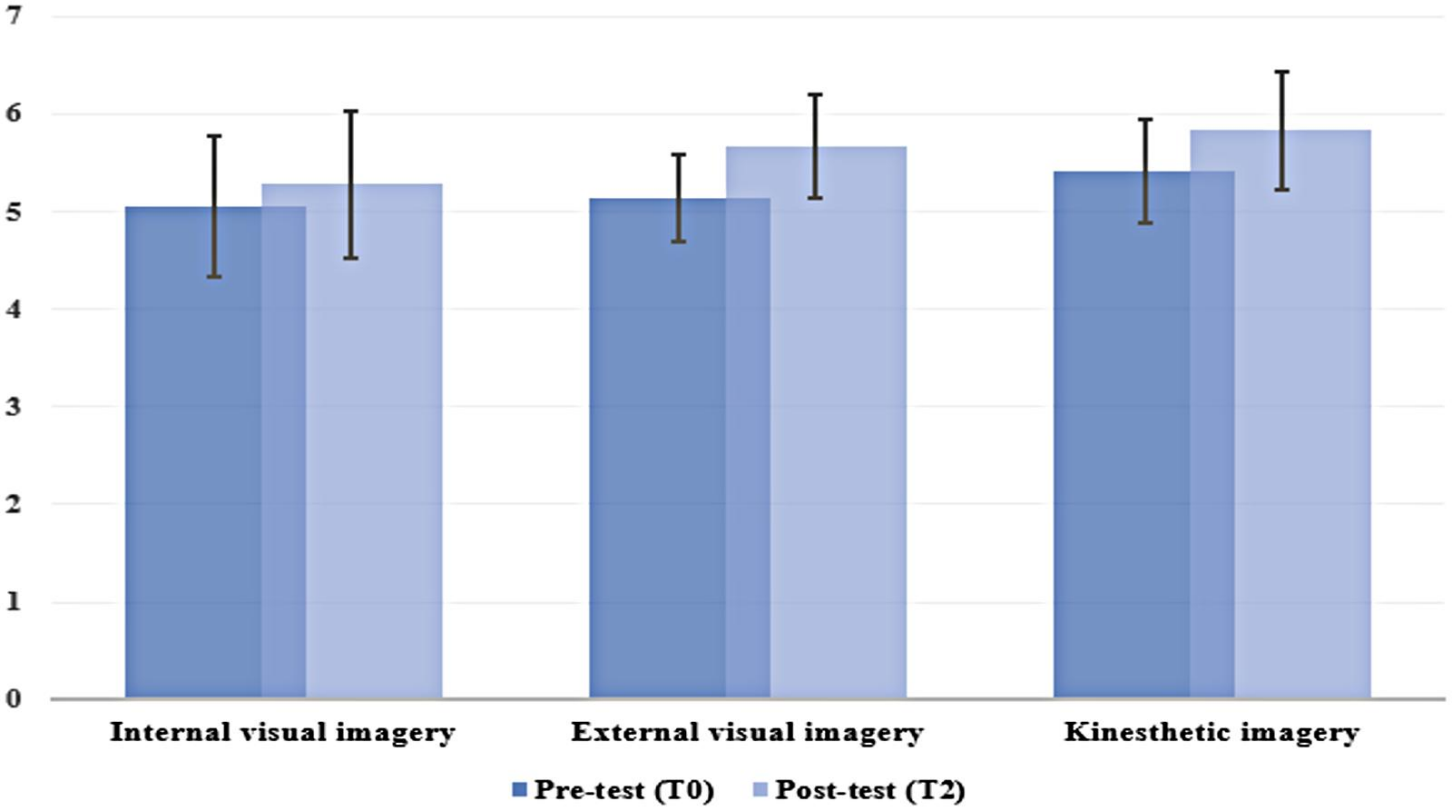
MIQ-3 scores by dimension from T0 to T2, with error bars representing standard deviation (SD) (*N* = 9).

MIQ-3 results showed increases in mean scores between the initial assessment (T0) and the final assessment (T2) for all three evaluated dimensions: internal visual imagery (5.06 → 5.28), external visual imagery (5.14 → 5.67), and kinesthetic imagery (5.42 → 5.83). The largest mean differences were observed for external visual imagery and kinesthetic imagery (Table 1, Figure 1).

**Table 1:** Descriptive MIQ-3 data by dimension (initial vs. final).

Dimension	Mean T0	SD_T0	CV%	Mean T2	SD_T2	CV%
Internal visual imagery	5.06	0.72	14.23	5.28	0.76	14.39
External visual imagery	5.14	0.44	8.56	5.67	0.53	9.35
Kinesthetic imagery	5.42	0.53	9.78	5.83	0.61	10.46

SD, standard deviation; CV, coefficient of variation. CV% was calculated as (SD/Mean)   ×   100 and rounded to two decimal places.

At T0, the mean values of the three dimensions were relatively similar, with the highest mean observed for kinesthetic imagery. The same pattern was maintained at T2, against a background of higher mean scores for all evaluated dimensions. The coefficients of variation remained within similar ranges across the two testing moments, without marked changes in group-level dispersion (Table 1).

### 3.2 Body aesthetics (EBAS)

**Figure 2:**
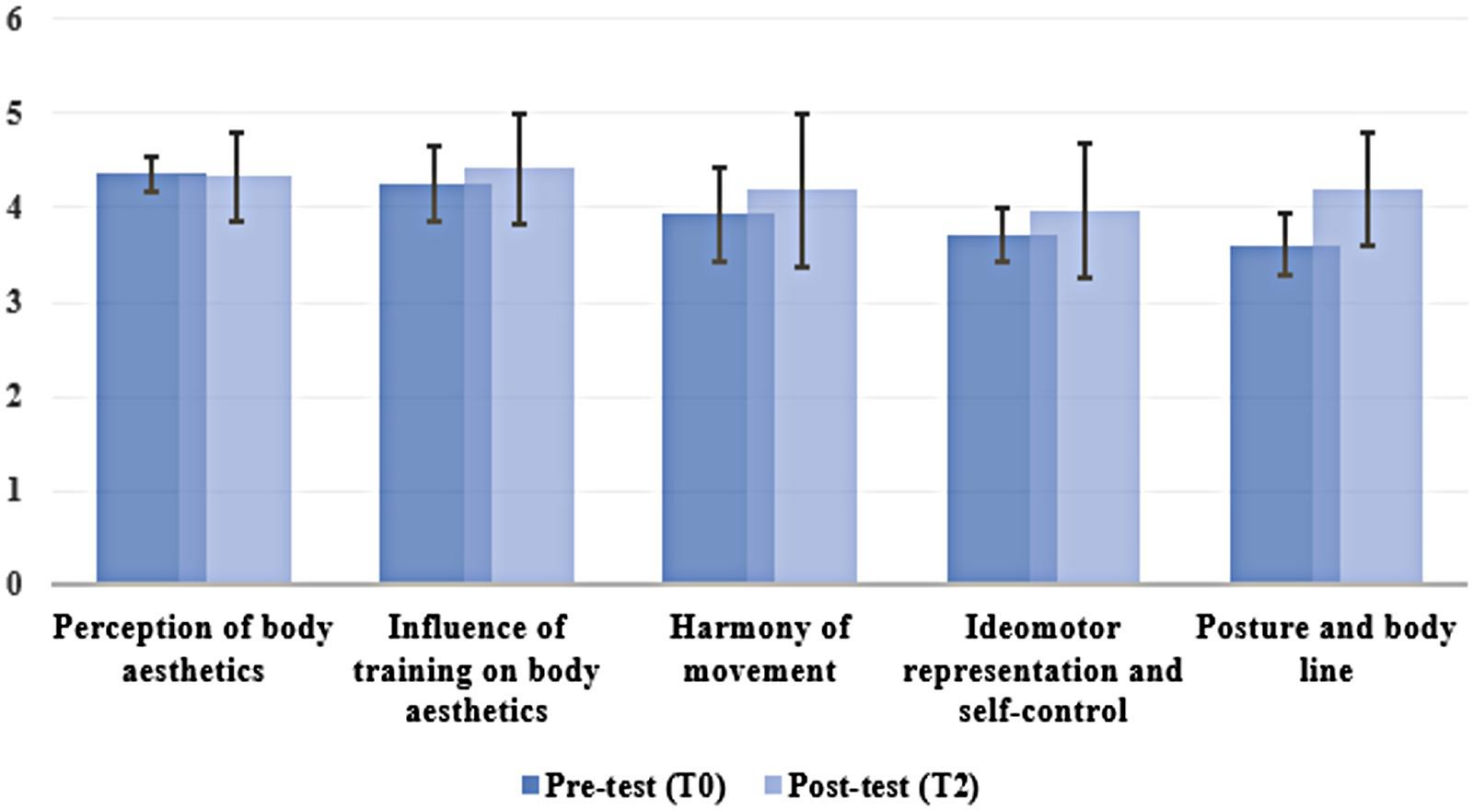
Mean EBAS scores by section from T0 to T2, with error bars representing standard deviation (SD) (*N* = 9).

EBAS results showed variations in mean scores between the initial assessment (T0) and the final assessment (T2) for most of the evaluated sections. Increases in mean values were observed for the influence of training on body aesthetics (4.25 → 4.42), harmony of movement (3.94 → 4.19), ideomotor representation and self-control (3.72 → 3.97), and posture and body line (3.61 → 4.19), whereas perception of body aesthetics showed similar values across the two time points (4.36 → 4.33) (Table 2, Figure 2).

**Table 2:** Descriptive EBAS data by section (initial vs. final).

Section	Mean T0	SD_T0	CV%	Mean T2	SD_T2	CV%
Section 1 – Perception of body aesthetics	4.36	0.18	4.13	4.33	0.48	11.09
Section 2 – Influence of training on body aesthetics	4.25	0.40	9.41	4.42	0.59	13.35
Section 3 – Harmony of movement	3.94	0.50	12.69	4.19	0.82	19.57
Section 4 – Ideomotor representation and self-control	3.72	0.29	7.80	3.97	0.72	18.14
Section 5 – Posture and body line	3.61	0.33	9.14	4.19	0.60	14.32

SD, standard deviation; CV, coefficient of variation. CV% was calculated as (SD/Mean)  ×  100 and rounded to two decimal places.

At T0, the highest mean was recorded for the section concerning perception of body aesthetics (4.36), and the lowest for the posture and body line section (3.61). At T2, the highest values were observed for the influence of training on body aesthetics (4.42), harmony of movement (4.19), and posture and body line (4.19), whereas the ideomotor representation and self-control section had a mean of 3.97 (Table 2). The coefficients of variation were higher at T2 than at T0 for all EBAS sections, increasing from 4.13% to 11.09% for perception of body aesthetics, from 9.41% to 13.35% for the influence of training on body aesthetics, from 12.69% to 19.57% for harmony of movement, from 7.80% to 18.14% for ideomotor representation and self-control, and from 9.14% to 14.32% for posture and body line (Table 2).

### 3.3 Whole-routine execution and motor control

The results regarding whole-routine performance showed increases in total score between the initial assessment (T0) and the final assessment (T2) for all 9 athletes, with individual differences ranging from +0.05 to +4.40 points. Detailed individual whole-routine values are provided in Supplementary Table S5.

**Figure 3:**
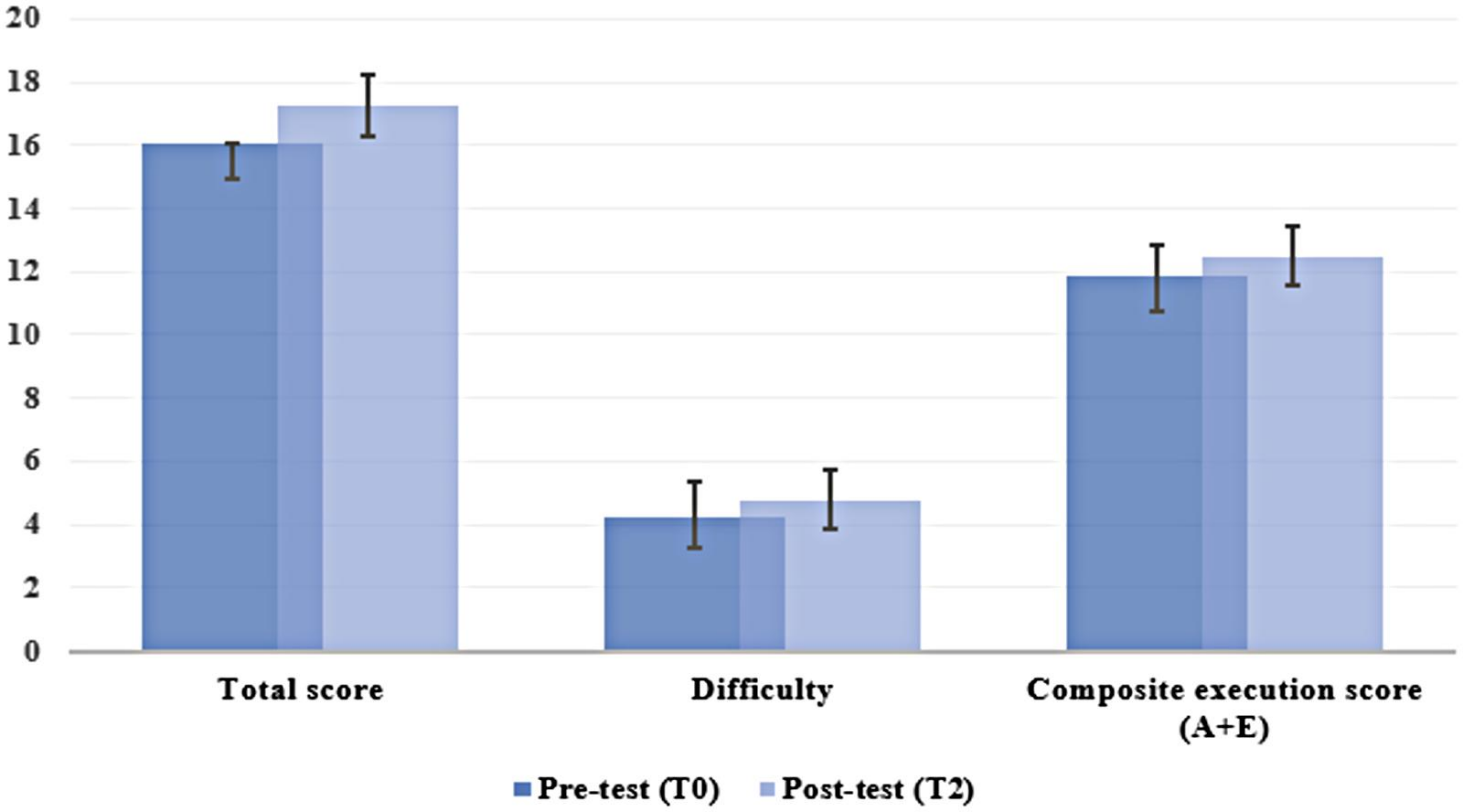
Mean changes in total score, difficulty, and composite execution score (A + E) from T0 to T2, with error bars representing standard deviation (SD) (*N* = 9).

At group level, the mean total score increased from 16.06 ± 1.10 to 17.26 ± 1.00 points. Over the same interval, mean difficulty increased from 4.27 ± 0.98 to 4.78 ± 0.92, and the mean composite execution score (A + E) increased from 11.86 ± 1.10 to 12.48 ± 0.93 (Table 3, Figure 3).

**Table 3:** Descriptive data for initial and final scores in whole routines with apparatus.

Component	Mean T0	SD_T0	CV% T0	Mean T2	SD_T2	CV% T2
Total score	16.06	1.10	6.83	17.26	1.00	5.81
Difficulty (D)	4.27	0.98	22.90	4.78	0.92	19.32
Composite execution score (A + E)	11.86	1.10	9.28	12.48	0.93	7.48

T0, initial assessment; T2, final post-intervention assessment; SD, standard deviation; CV, coefficient of variation; D, difficulty; A + E, composite execution score obtained by summing the artistic and technical execution components.

The coefficients of variation were lower at T2 than at T0 for total score, difficulty, and the composite execution score, decreasing from 6.83% to 5.81% for total score, from 22.90% to 19.32% for difficulty, and from 9.28% to 7.48% for the composite execution score (Table 3). Based on the relationship between realized difficulty and scripted difficulty, the mean difficulty realization rate increased from 64.66% to 72.82%.

### 3.4 Pre–post inferential analysis

Based on the results of the Shapiro–Wilk test, the global MIQ-3 score did not meet the normality criterion and was therefore analyzed using the Wilcoxon paired-samples test, whereas EBAS, difficulty (D), and the composite execution score (A + E) showed distributions compatible with normality and were analyzed using paired-samples t-tests.

At group level, the pre–post inferential analysis identified a statistically significant difference only for the global MIQ-3 score. The Wilcoxon paired-samples test indicated a significant difference between T0 and T2 (Z = −2.25, *p* = 0.024), associated with a large effect size (r = 0.75).

For EBAS, the T0–T2 mean difference was −0.24 and did not reach statistical significance [t(8) = −1.43, *p* = 0.189, Cohen's d = −0.48]. Similarly, the pre–post differences observed for difficulty and for the composite execution score (A + E) were not statistically significant. For difficulty, the T0–T2 mean difference was −0.51 [t(8) = −1.51, *p* = 0.167, Cohen's d = −0.51], whereas for A + E the T0–T2 mean difference was −0.62 [t(8) = −1.33, *p* = 0.218, Cohen's d = −0.45].

Overall, the inferential results indicated a statistically significant pre–post change only for ideomotor representation, whereas EBAS and the whole-routine performance components showed favorable descriptive changes that did not reach statistical significance. The corresponding statistical-output summary is provided in Supplementary Table S6.

### 3.5 Individual-level changes

The individual-level analysis showed heterogeneous pre–post changes across the three central dimensions investigated. For MIQ-3, 8 of the 9 athletes showed higher scores at T2 than at T0, and the Reliable Change Index (RCI) indicated reliable positive change in 3 athletes.

For EBAS, increases were observed in 5 athletes, stability in 1 athlete, and decreases in 3 athletes. RCI analysis indicated reliable positive change in 5 athletes, non-significant change in 2 athletes, and reliable negative change in 2 athletes.

For whole-routine performance, all 9 athletes recorded higher total scores at T2 than at T0, although the magnitude of change varied across participants. For whole-routine performance and its judged components, namely difficulty (D) and the composite execution score (A + E), RCI was not applied because, at each testing moment, the execution with the highest score out of two attempts was retained. Under these conditions, individual changes in these variables were interpreted descriptively on the basis of T2–T0 differences.

Detailed participant-level values and RCI results are provided in Supplementary Tables S7–S9.

### 3.6 Coefficient of harmony (CA) and weighted coefficient of harmony (CA-P)

The Coefficient of Harmony (CA) and the Weighted Coefficient of Harmony (CA-P), calculated on the basis of Total score, EBAS, and MIQ-3, showed a non-uniform pattern of change between T0 and T2. At individual level, CA increased in 3 athletes and decreased in 6 athletes, while CA-P also increased in 3 athletes and decreased in 6 athletes. However, the individual pattern was not identical for the two indicators: CA increased in J4_01, J3_03, and J2_01, whereas CA-P increased in J4_01, J3_01, and J2_01. Detailed individual CA and CA-*P* values are provided in Supplementary Table S10.

At group level, CA decreased from 0.62 ± 0.26 at T0 to 0.41 ± 0.25 at T2, with a mean difference of −0.21 (Table 4). This difference did not reach statistical significance (Z = −1.362, *p* = 0.173). Similarly, CA-P decreased from 0.63 ± 0.27 at T0–0.42 ± 0.25 at T2, with a mean difference of −0.21, also without statistical significance (Z = −1.362, *p* = 0.173).

**Table 4:** Group-level descriptive indicators for CA and CA-P at T0 and T2.

Indicator	T0 Mean ± SD	T2 Mean ± SD	Mean *Δ*
CA	0.62 ± 0.26	0.41 ± 0.25	−0.21
CA-P	0.63 ± 0.27	0.42 ± 0.25	−0.21

T0, initial assessment; T2, final post-intervention assessment; CA, Coefficient of Harmony; CA-P, Weighted Coefficient of Harmony; SD, standard deviation; *Δ* = T2–T0 difference.

These results indicate that the changes observed between T0 and T2 did not lead to a uniform increase in multidimensional harmony at group level. CA and CA-P therefore had a predominantly descriptive and exploratory value, suggesting a differentiated reorganization of the relationship among whole-routine performance, body aesthetics, and ideomotor representation.

### 3.7 Competitive performance and transfer

The competitive results showed variation across athletes both in the score obtained at the National Championship and in the best score recorded in the secondary competitions of the 2023 season (Table 5).

**Table 5:** Competitive performance (All-around) – national championship and best result in secondary competitions.

Subject	NC – AA score	NC – ranking position	Best secondary score
J4_01	29.950	5	36.450
J3_01	54.350	5	41.250
J3_02	47.300	9	37.416
J3_03	41.300	12	36.850
J3_04	54.300	4	54.250
J3_05	53.850	5	49.000
J3_06	48.450	7	36.650
J3_07	47.600	10	40.000
J2_01	46.950	14	42.450

Scores are expressed as the total score obtained in the All-Around (AA) event. The National Championship (NC) was used as the main reference competition of the season. “Best secondary score” represents the best score obtained by each athlete in the secondary competitions of the 2023 competitive season, excluding the National Championship result.

At the National Championship, All-Around scores ranged from 29.950 to 54.350 points, and ranking positions ranged from 4th to 14th place. In the secondary competitions, the best individual score ranged from 36.450 to 54.250 points (Table 5).

Spearman correlation analysis identified a positive association between CA_post and CA-P_post (*ρ* = 0.717, *p* = 0.030). By contrast, the correlations between CA_post and competitive performance did not reach statistical significance for the National Championship score (*ρ* = 0.250, *p* = 0.516), National Championship ranking position (*ρ* = 0.119, *p* = 0.761), or best secondary score (*ρ* = 0.017, *p* = 0.966). Similarly, the correlations between CA-P_post and competitive performance were not statistically significant for the National Championship score (*ρ* = 0.117, *p* = 0.765), National Championship ranking position (*ρ* = 0.424, *p* = 0.256), or best secondary score (*ρ* = 0.100, *p* = 0.798). The full correlation matrix is provided in Supplementary Table S11.

In the analyzed group, short-term transfer to competitive performance showed a variable pattern, with no significant associations between the synthetic indicators of the functional profile and competitive performance.

## 4 Discussion

The present study examined pre–post changes in ideomotor representation, body aesthetics, and motor control estimated through whole-routine performance during a personalized integrative training program in juvenile rhythmic gymnasts. Overall, at group level, the clearest change occurred in ideomotor representation, whereas body aesthetics and whole-routine performance showed favorable but statistically non-significant changes. Response variability across the group indicated that the three dimensions did not evolve synchronously during the monitored period. Given the small sample size, the exploratory design, and the absence of a control group, the findings should be interpreted cautiously and should not be generalized beyond the analyzed group.

### 4.1 Ideomotor representation: the clearest pre–post change

At group level, the global MIQ-3 score was the only variable that showed a statistically significant difference between T0 and T2 (Z = −2.25, *p* = 0.024), associated with a large effect size (r = 0.75). At the same time, the descriptive analysis by dimension indicated increases in mean scores for all three evaluated components, namely internal visual imagery (5.06 → 5.28), external visual imagery (5.14 → 5.67), and kinesthetic imagery (5.42 → 5.83), with the largest differences observed for external visual imagery and kinesthetic imagery. Taken together, these results indicate that self-reported movement imagery ability was the dimension that changed most clearly during the monitored period.

This finding is consistent with the literature describing motor imagery as a functional process involved in the anticipation, organization, and regulation of action, through mechanisms partly shared with those activated during actual movement execution (9, 10, 13). Within this framework, the increase in MIQ-3 scores may reflect a progressive clarification of the internal representation of the motor task, under conditions in which mental practice was systematically integrated into training and directly linked to execution. The findings are therefore compatible with the view that ideomotor representation may contribute to movement organization in juvenile rhythmic gymnastics, although this interpretation requires confirmation in larger controlled studies.

The comparatively clearer change in MIQ-3 scores may also be related to the structure of the training program. Motor imagery sequences were integrated directly with execution, in line with the principles of the PETTLEP model, which emphasize functional correspondence between imagery and the actual conditions of the task (17, 18, 21). In a discipline in which precision, synchronization, and expressiveness must be supported simultaneously, this proximity to the actual context of the routine may facilitate the consolidation of the internal representation of movement. Consistent with this, the literature shows that the combination of action observation and video observation with motor imagery may support the consolidation of movement representation and the refinement of execution (19, 20). In addition, MIQ-3 has been used as a general measure of movement imagery ability in athletic populations, although it is not specific to rhythmic gymnastics (35, 36).

From an applied perspective, this pattern may be relevant in the context of youth sport. In athletes who are still in the process of technical and cognitive consolidation, changes in the internal organization of movement may become detectable before a complete stabilization of execution or a more consistent aesthetic expression is achieved. This interpretation is also consistent with evidence suggesting that the development of motor imagery capacity in children and preadolescents is still ongoing and may be influenced by experience and specific practice (15). Under these conditions, mental practice may be viewed not only as a technique for supporting performance, but also as a possible means of consolidating the internal organization of action, with potential later transfer to execution stability and bodily expressiveness. Within the limits of the present study, ideomotor representation may constitute a useful component in the design of integrative interventions for juvenile rhythmic gymnasts.

### 4.2 Body aesthetics: favorable descriptive trends and heterogeneous change

Compared with ideomotor representation, body aesthetics showed a less uniform pattern of change. At group level, the global EBAS score indicated a modest increase, but the pre–post difference did not reach statistical significance (t = −1.43, *p* = 0.189). At the same time, the section-level analysis highlighted favorable changes especially for posture and body line (3.61 → 4.19), harmony of movement (3.94 → 4.19), and ideomotor representation and self-control (3.72 → 3.97), whereas the section referring to perception of body aesthetics remained relatively stable (4.36 → 4.33). These results suggest a favorable but statistically non-significant tendency in selected components of perceived body aesthetics, with a more heterogeneous pattern than that observed for MIQ-3.

This profile is compatible with the specific nature of the aesthetic dimension in rhythmic gymnastics. In this discipline, body aesthetics is not reduced to the outward appearance of movement, but expresses the level of integration among posture, amplitude, fluidity, expressiveness, and the coherent relationship with music and apparatus (2, 24). From this perspective, aesthetics does not represent an external addition to technique, but rather the visible form of a more complex functional organization in which motor control and expressive intention must become coherent within the whole routine (26). For this reason, one possible interpretation is that this dimension may require a longer period of consolidation than ideomotor representation, because it involves not only practice but also the gradual internalization of more subtle bodily, expressive, and musical landmarks.

In addition, the results may also be interpreted through the lens of the nonlinear character of motor learning. The literature from constraints-led and nonlinear pedagogy perspectives shows that adaptation to complex tasks does not evolve identically in all athletes, but depends on the interaction among task, environmental, and performer constraints, which favors different rhythms of reorganization of motor behavior (42, 43). In this sense, the heterogeneous EBAS response may reflect differentiated adaptation, influenced by the initial profile, expressive availability, postural stability, and the capacity to integrate artistic demands into technical execution, rather than a uniform response across the whole group.

Another important observation is that the EBAS dimensions that showed the clearest increases were precisely those most closely related to the functional organization of movement—posture and body line, harmony of movement, and self-control—rather than the more general global evaluation of body aesthetics. This suggests that changes may have been more visible in the structural and regulative components of bodily expression, whereas their integration into a more stable global aesthetic perception may require a longer period of consolidation. Such an interpretation is also consistent with the observation that, in complex motor tasks, different components of performance do not stabilize simultaneously, but may follow distinct trajectories of development and transfer (44).

Finally, it should also be taken into account that EBAS is a self-report instrument. Under these conditions, part of the variability observed at post-test may reflect not only changes in bodily expressiveness itself, but also a recalibration of internal criteria of judgment. As athletes become more aware of postural demands, body line, or the expressive coherence of the routine, it is possible that self-evaluation becomes more demanding, even under conditions of observable changes in execution. This interpretation remains cautious, but it may help explain why EBAS showed a more heterogeneous pattern than MIQ-3 in the analyzed group.

### 4.3 Motor control estimated through whole-routine performance: possible links with ideomotor representation

Although the clearest change was observed at the level of ideomotor representation, whole-routine performance also showed favorable descriptive changes, which was used in this study as an operational expression of motor control in a competitive context. At group level, the mean total score increased from 16.06 ± 1.10 to 17.26 ± 1.00 points, with increases observed across the sample. At the same time, the mean difficulty realization rate increased from 64.66% to 72.82%, which may indicate a more effective conversion of planned content into realized difficulty during execution.

At the inferential level, the changes in difficulty and in the composite execution score (A + E) did not reach statistical significance at group level (D: t = −1.51, *p* = 0.167; A + E: t = −1.33, *p* = 0.218). Therefore, these changes should be interpreted primarily as favorable descriptive trends rather than as statistically confirmed effects. However, in rhythmic gymnastics, where small point differences may influence the final ranking, and where performance depends simultaneously on difficulty, execution stability, and artistic expressiveness, such directional changes may still retain practical relevance, even in the absence of a statistically significant group-level difference (1, 2, 4).

In this context, the observed improvement in ideomotor representation may be interpreted as a process that could support more efficient organization of execution, without this transfer being automatic or immediate. The literature on movement representation suggests that effective motor performance relies on functional cognitive structures of action, and that modification of these structures may precede the stabilization of behavior observable in execution (12). From this perspective, the increases observed in MIQ-3 may reflect a progressive clarification of the internal representation of the motor task, namely of the technical sequences, the succession of elements, and the body–apparatus relationships, with the potential to facilitate action anticipation and organization (11, 20, 45).

At the same time, the results suggest that this relationship between ideomotor representation and execution is not expressed uniformly and simultaneously across the observed outcomes. Although MIQ-3 scores increased more consistently, the magnitude of the changes observed in realized difficulty and in the composite execution score (A + E) was more variable. Such a configuration is consistent with the literature on nonlinear motor learning and constraints-led perspectives, according to which adaptation in complex tasks depends on the interaction among task demands, performer characteristics, and practice context, whereas the reorganization of coordination and control may follow different and non-synchronous trajectories (42, 43, 46–49). Consequently, it is plausible that, in the present group, improvement in ideomotor representation represented only one possible condition supporting the reorganization of execution, without implying that this reorganization had already reached the same level of stabilization across all performance components.

This interpretation is also supported by the literature on the implementation of mental practice. Motor imagery interventions tend to be more effective when they are adapted to the athlete, integrated with physical practice, and associated with content relevant to the real task (10, 50). In the present study, mental practice was not used as an isolated module, but was inserted systematically into the training structure, before key segments, whole-routine executions, and simulations, in accordance with PETTLEP logic. Therefore, it is reasonable to assume that the intervention may have contributed first to the internal organization of movement, whereas transfer toward realized difficulty and execution quality may have depended on technical stability, regulatory possibilities, and the initial profile of each athlete.

At the same time, the relationship between changes in representation and changes in execution should be interpreted with caution. The literature shows that variability observed during learning does not always have the same functional meaning and that the effects of an intervention depend on task nature and on the performer's stage of learning (44). In a discipline such as rhythmic gymnastics, body–apparatus–music coordination simultaneously requires precision, temporal control, and expressive continuity, which makes it plausible that adaptations at the level of ideomotor representation transfer to execution at different rates, depending on the athlete's initial profile, technical stability, and capacity to integrate the demands of the whole routine (1, 2, 51). From an applied perspective, this suggests that progress should not be evaluated exclusively through the final point difference, but also through the dynamics of the relationship between the internal organization of action and the stabilization of execution itself.

### 4.4 Coefficient of harmony, weighted coefficient of harmony, and exploratory profile reorganization

The Coefficient of Harmony (CA) and the Weighted Coefficient of Harmony (CA-P), used in this study as exploratory indicators of the reciprocal support among MIQ-3, EBAS, and Total score, showed similar decreases in mean values between T0 and T2 at group level (CA: 0.62 ± 0.26 → 0.41 ± 0.25; CA-P: 0.63 ± 0.27 → 0.42 ± 0.25), without reaching the threshold of statistical significance (Z = −1.362, *p* = 0.173). These results indicate that the changes observed during the monitored period did not lead to a uniform increase in multidimensional harmony at group level.

These indicators should be interpreted with caution, because they do not express the absolute level of performance, but rather the internal configuration of the relationship among the analyzed dimensions. Therefore, lower CA or CA-*P* values do not automatically signify regression, but may reflect a temporary imbalance in the relationship among components when one dimension evolves more clearly than the others. This interpretation is consistent with the literature showing that variability arising during learning should not automatically be equated with flexibility or superior progress, but must be judged in relation to task demands and to the type of reorganization required (44, 52). In the present study, this interpretation is plausible especially because ideomotor representation showed the clearest pre–post change, whereas body aesthetics and whole-routine performance showed favorable but statistically non-significant changes. Such a reading is compatible with the literature describing motor performance as the result of a functional organization of action, supported by dynamic relationships among representation, control, and execution, relationships that do not necessarily stabilize simultaneously during learning (12).

From this perspective, CA and CA-P are useful primarily as descriptive and exploratory indicators that summarize the configuration of the relationship among ideomotor representation, body aesthetics, and whole-routine performance during the monitored period, and less as central outcomes of the study. Given the small sample size and the exploratory design, these indicators should be regarded as supportive descriptors of the pattern observed at group level rather than as independent evidence of intervention efficacy.

### 4.5 Competitive performance and the limits of short-term transfer

With regard to competitive performance, the results showed substantial variability among athletes. At the National Championship, All-Around scores ranged from 29.950 to 54.350 points, whereas ranking positions ranged from 4th to 14th place. In the secondary competitions, the best individual score ranged from 36.450 to 54.250 points. These data indicate that, although the athletes followed the same general intervention logic, the competitive expression of progress remained heterogeneous, which is expected in a discipline in which the final result depends on the simultaneous integration of difficulty, execution, the artistic component, and the competition context.

The correlational analysis showed that the only statistically significant association was that between CA_post and CA-P_post (*ρ* = 0.717, *p* = 0.030), which indicates convergence between the two synthetic indicators in describing the functional profile at post-test. By contrast, the relationships between these indicators and competitive performance did not reach statistical significance, indicating that the internal configuration described by CA and CA-P was not associated with an immediate competitive advantage in the present sample.

This result suggests that transfer to competitive performance is likely mediated by additional factors. In rhythmic gymnastics, competitive outcome depends not only on the quality of the internal organization of movement, but also on the absolute level of difficulty, the stability of execution on the day of competition, the particularities of composition, the capacity to manage the competitive context, and judging (1, 2, 4). Consequently, it is plausible that the changes observed during the study period are more easily detected initially at the level of ideomotor representation and of the relationship among components than in the competitive score itself.

Viewed in relation to the broader issue of performance in young athletes, this result suggests that adaptations associated with an integrative program do not necessarily transfer immediately and linearly to competitive performance. In juvenile athletes, the internal development of movement organization and the stabilization of competitive expression may follow different rhythms, and competitive performance may remain, in the short term, more sensitive to contextual variables than to functional reorganization that is still in the process of consolidation.

### 4.6 Practical implications and study limitations

The results suggest the potential usefulness of an integrated approach in the preparation of juvenile rhythmic gymnasts and point to the relevance of explicitly including mental practice and ideomotor representation in the structure of the intervention. From an applied perspective, this observation suggests that the internal organization of action may constitute a useful entry point for supporting subsequent adaptations in execution. In the context of youth sport, such an orientation is also consistent with the literature emphasizing the importance of designing the learning environment and adjusting task constraints to the athlete's developmental level in order to foster functional skill acquisition and individualized progress (42, 43, 53). From this perspective, the potential value of the intervention lies not only in the content of the exercises used, but also in the way the structure of practice was adapted to the developmental level, initial profile, and the need for progressive integration of motor, mental, and expressive demands.

For coaches, this perspective offers a more nuanced framework for interpreting progress. Progress in ideomotor representation may occur without being immediately transferred to bodily expressiveness or execution stability. Similarly, favorable changes in realized difficulty may coexist with a slower evolution of the aesthetic dimension. Under these conditions, the simultaneous evaluation of whole-routine execution, ideomotor representation, and body aesthetics may provide additional reference points for individualizing the training process, rather than relying exclusively on the final score, in line with recommendations regarding the integrated and adapted use of motor imagery in sport (10, 50).

At the same time, the findings must be interpreted in relation to the limitations of the study. The small sample size, the heterogeneous composition of the group, and the relatively short duration of the intervention limit the inferential power of the analyses and the degree of generalization of the results. Competitive performance was analyzed under ecological conditions, so the contextual variables specific to competition could not be fully controlled. In addition, the absence of a control group does not allow complete separation of changes specifically attributable to the intervention from the influence of regular training, maturation, and the dynamics specific to the competitive season. For variables derived from the evaluation of the whole routine, measurement reliability is also more difficult to estimate than in the case of questionnaires, and the fact that, at each testing moment, the execution with the highest score out of two attempts was retained limits the assessment of intra-individual variability and of performance stability across attempts. Another important limitation derives from the use of a self-report instrument for body aesthetics, in which case part of the variability in responses may reflect changes in internal criteria of judgment, and not exclusively changes in bodily expressiveness itself.

Overall, these data suggest that an integrative perspective may provide useful reference points for individualizing the training process in juvenile rhythmic gymnasts, but the present findings should be regarded as exploratory and require confirmation in larger, controlled studies.

## 5 Conclusions

The results of the present study suggest that, in the group analyzed, pre–post changes observed during a personalized integrative training program were most evident for ideomotor representation, while body aesthetics and motor control estimated through whole-routine performance showed favorable but less uniform developments. In the analyzed group, ideomotor representation was the only variable that showed a statistically significant change at group level, indicating that mental practice and motor imagery may represent relevant components in the organization and regulation of action in juvenile rhythmic gymnasts. Body aesthetics and whole-routine performance also showed favorable but less uniform developments, suggesting that the relationship among ideomotor representation, motor control, and aesthetic expression may evolve at different rates across athletes.

In this context, the Coefficient of Harmony (CA) and the Weighted Coefficient of Harmony (CA-P) had mainly descriptive utility, highlighting the individualized nature of the reorganization of the functional profile. From an applied point of view, the results suggest the potential usefulness of an integrated and personalized approach in the preparation of juvenile rhythmic gymnasts, while remaining limited by the exploratory design, small sample size, and absence of a control group. Future research should examine these relationships in larger samples, in differentiated subgroups, and over longer intervention intervals, in order to clarify the extent to which this reorganization is transferred to execution stabilization and to competitive performance.

## Data Availability

The datasets generated and analyzed during this study are not publicly available due to ethical and confidentiality considerations related to minor participants. De-identified data supporting the findings may be made available by the corresponding author upon reasonable request, subject to applicable ethical and institutional requirements.
