# Supplementary material for: Ideomotor representation, motor control, and body aesthetics in juvenile rhythmic gymnasts: between reciprocal support and differentiated trajectories within a personalized training program
Source: Front Sports Act Living. 2026 Jul 20;8:1874791. doi: 10.3389/fspor.2026.1874791 (PMC13429731; doi:10.3389/fspor.2026.1874791)
Supplement: Supplementary file 1 [file Supplementaryfile1.docx]

**Supplementary Material: Intervention Structure, Assessment Instruments, Computation Procedures, and Supplementary Results**

**1 Supplementary Data**

This supplementary file includes eleven supplementary tables and two supplementary methodological appendices:

- Supplementary Table S1. Detailed structure of the 14-week personalized integrative training program.
- Supplementary Table S2. Exercise core and coding key of the personalized integrative training program.
- Supplementary Table S3. Baseline personalization matrix used to individualize the integrative training program.
- Supplementary Table S4. Whole-routine models used for assessment and intervention transfer.
- Supplementary Appendix S1. Assessment instruments: EBAS and MIQ-3 administration and scoring procedures.
- Supplementary Appendix S2. Detailed computation procedure for the Coefficient of Harmony (CA) and the Weighted Coefficient of Harmony (CA-P).
- Supplementary Table S5. Individual whole-routine results at T0 and T2.
- Supplementary Table S6. Detailed inferential statistics for pre–post comparisons.
- Supplementary Table S7. Individual evolution of MIQ-3, EBAS, and total score between T0 and T2.
- Supplementary Table S8. Individual progress analysis for MIQ-3 using the Reliable Change Index (RCI).
- Supplementary Table S9. Individual progress analysis for EBAS using the Reliable Change Index (RCI).
- Supplementary Table S10. Individual CA and CA-P values at T0 and T2.
- Supplementary Table S11. Correlations between CA, CA-P, and competitive performance.

**Supplementary Table S1. Detailed structure of the 14-week personalized integrative training program**

The personalized integrative training program was embedded in a 52-week annual training plan for the 2023 competitive season, which included preparatory, pre-competitive, competitive, and transition phases. Only the 14-week intervention window, extending from MZC2 to the early part of MZC5, was monitored through pre–post assessments (T0–T2). Subsequent competition results were used only to examine the ecological transfer of the intervention. The program was organized around three central components—motor control, body aesthetics, and ideomotor representation—and followed a progressive “mosaic” structure, in which mental practice, technical execution, aesthetic refinement, whole-routine work, and individualized feedback were combined within the same training process.

Personalization was based on each athlete’s T0 profile, which included indicators of whole-routine performance, difficulty realization rate, composite execution score (A+E), EBAS score, and MIQ-3 score. According to this profile, the emphasis placed on motor control, body aesthetics, and ideomotor representation was classified as high, medium, or maintenance for each athlete. These priorities guided the selection and combination of the CM, EC, and RI exercise blocks within the weekly “mosaic” structure, without creating separate individualized programs outside the regular training process. The monitored intervention is summarized below.

| **Period / weeks** | **Mesocycle and training phase** | **Main objective** | **Motor control content** | **Body aesthetics content** | **Ideomotor representation content** | **Whole-routine integration and feedback** |
| --- | --- | --- | --- | --- | --- | --- |
| Weeks 1–4 / February | MZC2 – accumulation and standardization | Establishing the foundations of motor control and body aesthetics; initiating ideomotor representation; consolidating basic technical work. | Assisted and unassisted balance exercises; postural axis control; stability in basic balance positions; introductory object-control tasks; simple throws and catches. | Basic posture and body-line work; barre-based exercises; arm-position control; elementary dance steps; controlled amplitude and fluidity. | Introductory visualization of the whole routine and short technical sequences; basic mental rehearsal before selected technical tasks; initial use of music-supported imagery. | Short routine fragments of approximately 30–60 s; mental rehearsal placed before key technical sequences; permanent postural correction; feedback focused on alignment, stability, and basic execution quality. |
| Weeks 5–8 / March | MZC3 – consolidation and integration into routine fragments | Consolidating motor control and body aesthetics and increasing integration into routine fragments. | Transfer from assisted to free execution; balance consistency; object-control precision; stabilization of throws, catches, and body–apparatus coordination. | Fluid transitions between difficulties; expressive arm and trunk movement; dance-step coherence; refinement of movement continuity. | Visualization of difficulty sequences synchronized with music; mental rehearsal of problematic fragments; use of the real starting position and task-specific imagery. | Increased use of partial routines and integrated fragments; RI→execution sequences; corrections individualized according to the T0 profile; feedback directed toward recurrent technical and artistic errors. |
| Weeks 9–12 / April | MZC4 – refinement, integration, and simulations | Refining execution quality, artistic integration, and stability under increasingly competition-like conditions. | Work on critical throws and catches; control under loss of visual contact; combinations involving axis changes; stabilization of difficulty realization and apparatus handling. | Artistic combinations on musical themes; expressiveness, body line, and musical coherence; refinement of transitions and expressive continuity. | Whole-routine and segment imagery with music and real timing; kinesthetic imagery; PETTLEP-based imagery including task, timing, learning, and emotional components. | Whole-routine executions and competition simulations; video-supported correction; routine-start stabilization; short feedback loops focused on penalties, rhythm, execution quality, and expressive coherence. |
| Weeks 13–14 / early May | MZC5 – pre-competitive fine-tuning until T2 | Stabilizing execution under competition-like conditions; controlled reduction of volume while preserving specific intensity; final assessment. | Maintenance of difficulty execution and apparatus-control precision; targeted correction of unstable elements; reduced volume of isolated correction work. | Maintenance of body line, posture, transitions, and expressive quality; minimal corrections focused on details relevant to the competitive routine. | PETTLEP imagery before whole-routine execution; emotional regulation imagery; real-time and music-supported visualization of the routine. | Competition simulations and whole-routine executions; immediate post-execution feedback; controlled pre-competitive adjustment; final assessment at T2. |

**Note.** CM = motor control; EC = body aesthetics; RI = ideomotor representation; MZC = mesocycle; T0 = initial assessment; T2 = final post-intervention assessment. The monitored intervention covered 14 weeks, with four weekly sessions and approximately 11 h of training per week, totaling 154 h. Of this volume, 33 h were allocated to motor control, 31 h to body aesthetics, and 14.5 h to ideomotor representation. The remaining time was distributed among specific technical preparation, whole-routine executions, simulations, and individualized feedback/correction sequences. Together, the three central components accounted for 78.5 h, representing approximately 51.0% of the monitored volume and 26.37% of the annual planned training volume (584 h). The exercise core used for methodological traceability comprised CM01–CM20, RI01–RI09, and EC01–EC13. The “mosaic” structure refers to the functional combination of these components within the same training session, for example RI→CM, EC→CM, RI→whole routine, or CM→whole routine followed by brief individualized feedback. Photographic materials and identifiable visual records were not included in the supplementary material.

The coded exercise core used in the personalized integrative training program is presented in Supplementary Table S2. The coding system was used for methodological traceability and for linking the weekly “mosaic” structure to the three central intervention components: motor control, ideomotor representation, and body aesthetics.

**Supplementary Table S2.** Exercise core and coding key of the personalized integrative training program

| **Code** | **Exercise no.** | **Exercise / task description** | **Component** |
| --- | --- | --- | --- |
| CM01 | Ex.110 | Split balance on the whole foot, with hand assistance and barre support | Motor control |
| CM02 | Ex.111 | Split balance on the whole foot, without hand assistance and with barre support | Motor control |
| CM03 | Ex.112 | Side split balance on the whole foot, with hand assistance and barre support | Motor control |
| CM04 | Ex.113 | Side split balance on the whole foot, without hand assistance and with barre support | Motor control |
| CM05 | Ex.114 | Panché balance on the whole foot, with barre support | Motor control |
| CM06 | Ex.115 | Ring balance on the whole foot, with barre support | Motor control |
| CM07 | Ex.116 | Side split balance on the whole foot, performed on the carpet, with or without hand assistance | Motor control |
| CM08 | Ex.117 | Panché balance on the whole foot, performed on the carpet | Motor control |
| CM09 | Ex.118 | Side split balance on the whole foot, with the support foot placed on a yoga block | Motor control |
| CM10 | Ex.119 | Panché balance on the whole foot, with the support foot placed on a yoga block | Motor control |
| CM11 | Ex.120 | Simple high throw with apparatus, using the dominant hand, followed by catch | Motor control |
| CM12 | Ex.121 | Simple high throw with apparatus, using the non-dominant hand, followed by catch | Motor control |
| CM13 | Ex.122 | High throw without hand contact, performed during rotation/pre-acrobatic movement, followed by catch | Motor control |
| CM14 | Ex.123 | High throw with the dominant hand, loss of visual contact, two vertical rotations under the throw, followed by catch | Motor control |
| CM15 | Ex.124 | High throw with the dominant hand, loss of visual contact, combined rotations with axis change under the throw, followed by catch | Motor control |
| CM16 | Ex.125 | Body difficulty elements of the jump/leap type performed with specific apparatus handling | Motor control |
| CM17 | Ex.126 | Body difficulty elements with rotation/pivots performed with specific apparatus handling | Motor control |
| CM18 | Ex.127 | Balance difficulty elements performed with specific apparatus handling | Motor control |
| CM19 | Ex.128 | Apparatus difficulty combinations performed without hand contact and/or without visual contact | Motor control |
| CM20 | Ex.129 | Complete individual routine with apparatus | Motor control |
| RI01 | Ex.130 | Whole-routine visualization in the starting position, with music in the background | Ideomotor representation |
| RI02 | Ex.131 | Visualization of difficulty sequences synchronized with music | Ideomotor representation |
| RI03 | Ex.132 | Visualization of technical apparatus elements, with emphasis on trajectory and catch | Ideomotor representation |
| RI04 | Ex.133 | Mental rehearsal of a combination of three to four body and technical elements | Ideomotor representation |
| RI05 | Ex.134 | Mental rehearsal of apparatus throws, with emphasis on correct catching | Ideomotor representation |
| RI06 | Ex.135 | Mental rehearsal of an artistic sequence, role, or narrative theme | Ideomotor representation |
| RI07 | Ex.136 | Visualization in the specific competition starting position, with apparatus in hand; PETTLEP-integrated task | Ideomotor representation |
| RI08 | Ex.137 | Visualization with emphasis on kinesthetic sensations, including arm tension and contact with the apparatus; PETTLEP-integrated task | Ideomotor representation |
| RI09 | Ex.138 | Visualization with emphasis on the emotional component, including audience presence and performance emotion; PETTLEP-integrated task | Ideomotor representation |
| EC01 | Ex.139 | First-position plié with correct arm posture, performed at the barre | Body aesthetics |
| EC02 | Ex.140 | Relevé at the barre with arm movement through the five fundamental positions of classical ballet | Body aesthetics |
| EC03 | Ex.141 | Arabesque at the barre with maintenance of the arm–leg line | Body aesthetics |
| EC04 | Ex.142 | Dance-step sequence with amplitude variations, performed on the carpet | Body aesthetics |
| EC05 | Ex.143 | Fluid transitions between two difficulties, with emphasis on elegance of movement | Body aesthetics |
| EC06 | Ex.144 | Classical-dance-inspired arm movements combined with diagonal travelling steps | Body aesthetics |
| EC07 | Ex.145 | Interpretation of an emotion through a dance-step sequence | Body aesthetics |
| EC08 | Ex.146 | Interpretation of a character through body elements and gestures | Body aesthetics |
| EC09 | Ex.147 | Artistic combination performed on a given musical theme | Body aesthetics |
| EC10 | Ex.148 | Seated position with one extended leg supported on a yoga block and toe extension using an elastic band | Body aesthetics |
| EC11 | Ex.149 | Seated position with bent legs and backward palm support; feet supported on a yoga block with progressive downward pressure of the knees over the split position | Body aesthetics |
| EC12 | Ex.150 | Kneeling position with support on a yoga block at the knees and toes, maintaining the over-toe position | Body aesthetics |
| EC13 | Ex.151 | Standing over the toes with the back to the barre; forward trunk flexion toward the legs with backward arm support on the barre, maintaining the position | Body aesthetics |

**Note.** CM = motor control; RI = ideomotor representation; EC = body aesthetics. The exercise numbers refer to the internal exercise catalogue used in the research protocol. The complete catalogue included 151 exercises; however, only Ex.110–Ex.151 constituted the coded intervention core directly associated with the three central components analyzed in the present article: motor control, ideomotor representation, and body aesthetics. Exercises Ex.1–Ex.109 represented general warm-up, technical support, mobility, and preparatory exercises and were not included in this supplementary table. Photographic materials and identifiable visual records were not included in the supplementary material.

**Supplementary Table S3. Baseline personalization matrix used to individualize the integrative training program**

| **Athlete code** | **Competitive category** | **Motor control priority** | **Body aesthetics priority** | **Ideomotor representation priority** | **Main individualized focus** |
| --- | --- | --- | --- | --- | --- |
| J4_01 | Junior IV | Maintenance | High | High | Clarification of movement representation from short sequences to the whole routine; basic posture and expressiveness; maintenance of motor-control transfer. |
| J3_01 | Junior III | Maintenance | Medium | Medium | Consolidation of expressiveness and coherence with music; maintenance of motor control with reduction of specific execution errors. |
| J3_02 | Junior III | High | Maintenance | Maintenance | Improvement of difficulty realization rate through stabilization of key elements and apparatus handling. |
| J3_03 | Junior III | High | Medium | Medium | Reduction of major execution errors and improvement of execution consistency; refinement of body lines in dominant elements. |
| J3_04 | Junior III | Maintenance | Maintenance | Maintenance | General maintenance and fine optimization of execution quality and stability in a pre-competitive context. |
| J3_05 | Junior III | High | High | Medium | Stabilization of elements with low difficulty realization rate; posture, body line, and transitions; motor imagery for problematic sequences. |
| J3_06 | Junior III | High | Maintenance | Maintenance | Reduction of execution deductions through postural control and apparatus precision; maintenance of aesthetic and ideomotor components. |
| J3_07 | Junior III | High | High | Medium | Improvement of stability and apparatus precision; expressiveness and quality of dance steps; maintenance of ideomotor representation. |
| J2_01 | Junior II | High | Maintenance | Maintenance | Control of execution quality at competitive intensity, with emphasis on fatigue management and precision. |

**Note.** Priorities were established from the T0 profile of each athlete and were used to guide the emphasis placed on motor control, body aesthetics, and ideomotor representation within the weekly “mosaic” structure. “Maintenance” indicates that the component was preserved and integrated into the program without additional emphasis beyond the planned training logic.

Whole-routine performance was assessed using the individual competitive routine with apparatus selected for each athlete and kept constant between T0 and T2. The full FIG symbolic notation was used internally for judging and methodological traceability, but only a synthetic description of the routine models is reported here to preserve clarity and avoid excessive technical detail in the supplementary file.

**Supplementary Table S4.** Whole-routine models used for assessment and intervention transfer

| **Athlete code** | **Competitive category** | **Apparatus** | **Scripted difficulty / planned D (pts.)** | **Main routine content represented in the model** |
| --- | --- | --- | --- | --- |
| J4_01 | Junior IV | Rope | 5.50 | Dynamic element with rotation, body waves, arabesque balance, dance-step series, panché balance, split jumps, apparatus difficulties, pivot elements, and complete individual routine structure. |
| J3_01 | Junior III | Ribbon | 7.80 | Multiple apparatus difficulties, fouetté dynamic balance, split leap with trunk extension and scissor action, panché balance, dynamic elements with rotations, dance-step series, pivot elements, and whole-routine integration. |
| J3_02 | Junior III | Rope | 6.90 | Apparatus difficulties, dance-step series, split leap with trunk extension and scissor action, panché pivot, dynamic elements with rotations, ring-form split jump, panché balance, fouetté dynamic balance, body wave, and pivot elements. |
| J3_03 | Junior III | Rope | 5.50 | Dynamic element with rotation, apparatus difficulties, dance-step series, ring-form split jump, sagittal split pivot with hand assistance, panché balance, body waves, fouetté dynamic balance, and panché pivot. |
| J3_04 | Junior III | Ribbon | 5.80 | Panché balance, body wave, apparatus difficulties, dance-step series, arabesque pivot, ring-form split jump, fouetté dynamic balance, lateral body wave, panché pivot, and expressive musical effects. |
| J3_05 | Junior III | Ball | 8.70 | Fouetté dynamic balance, apparatus difficulties, split leap with trunk extension and scissor action, body wave, dynamic elements with rotations, panché balance, dance-step series, panché pivot, split jump with trunk extension, and expressive musical effects. |
| J3_06 | Junior III | Ribbon | 6.80 | Apparatus difficulties, dance-step series, panché balance, split leap with trunk extension and scissor action, fouetté dynamic balance, dynamic elements with rotations, ring jump, panché pivot, and expressive musical effects. |
| J3_07 | Junior III | Ball | 5.80 | Apparatus difficulties, panché balance, dynamic elements with rotations, panché pivot, fouetté dynamic balance, dance-step series, split jump, split leap with scissor action, sagittal split pivot with hand assistance, and expressive musical effects. |
| J2_01 | Junior II | Ball | 6.90 | Apparatus difficulties, panché balance, dynamic elements with rotations, panché pivot, fouetté dynamic balance, dance-step series, split jump with trunk extension, split leap with scissor action, attitude pivot, and ring balance without hand assistance. |

**Note.** The full FIG symbolic notation was used internally for the construction and scoring of the whole routines. DA = apparatus difficulty; R = dynamic elements with rotation; D = dynamic changes in the symbolic routine sheets; E = expressive or musical effects created through the body or apparatus. The notation D used in this legend should not be confused with the planned difficulty score reported in the table. The same routine model was retained for each athlete between T0 and T2 to support intra-individual comparability. The table reports a synthetic description only; full symbolic sheets were not included in order to maintain readability and avoid excessive technical detail.

**Supplementary Appendix S1. Assessment instruments: EBAS and MIQ-3 administration and scoring procedures**

**S1.1. Evaluation of Body Aesthetics in Sports (EBAS)**

The Evaluation of Body Aesthetics in Sports (EBAS) was developed within the present research to assess perceived body aesthetics in the specific context of rhythmic gymnastics. The instrument was designed for athletes involved in aesthetic sports and addresses perceived body aesthetics, movement harmony, posture, motor control, and the perceived influence of training on aesthetic execution.

The questionnaire was administered in Romanian. The English version below is provided for reporting purposes.

**Instructions and response scale**

Participants were instructed to read each statement and indicate their level of agreement on a 5-point Likert scale:

1 = strongly disagree

2 = partially disagree

3 = neutral

4 = partially agree

5 = strongly agree

Higher scores indicate a more favorable perception of body aesthetics, movement harmony, posture, and aesthetic quality of execution. The instrument does not include reverse-worded items.

**Appendix Table S1.1.** EBAS instrument structure

| **Section** | **Thematic focus** | **Items** |
| --- | --- | --- |
| Section 1 | Perception of body aesthetics | Items 1–4 |
| Section 2 | Influence of training on body aesthetics | Items 5–8 |
| Section 3 | Harmony of movement | Items 9–12 |
| Section 4 | Ideomotor representation and self-control | Items 13–16 |
| Section 5 | Posture and body line | Items 17–20 |

In the present article, EBAS was treated as a global scale. The five sections were used descriptively to characterize the response profile and were not analyzed as independent psychometric subscales.

**Appendix Table S1.2.** EBAS items

| **Section** | **Item no.** | **Item wording** |
| --- | --- | --- |
| Perception of body aesthetics | 1 | I am satisfied with the way my body appears aesthetically during competitions. |
| Perception of body aesthetics | 2 | I am confident that my movements reflect pleasant and correct body aesthetics in routines. |
| Perception of body aesthetics | 3 | I believe that body aesthetics is as important as technical abilities in rhythmic gymnastics. |
| Perception of body aesthetics | 4 | The appearance of my body influences my self-confidence during training and competitions. |
| Influence of training on body aesthetics | 5 | My coach helps me correct body positions in order to achieve optimal aesthetics. |
| Influence of training on body aesthetics | 6 | Ballet exercises improve my body aesthetics during rhythmic gymnastics routines. |
| Influence of training on body aesthetics | 7 | Training exercises are designed to improve the aesthetics of my posture and movements. |
| Influence of training on body aesthetics | 8 | I consider that motor control training positively influences the aesthetics of my movements. |
| Harmony of movement | 9 | I believe that my movements are harmonious when they are well choreographed. |
| Harmony of movement | 10 | I imagine how the movements are connected to create a harmonious flow. |
| Harmony of movement | 11 | I feel more confident in performing routines when the movements are harmonious. |
| Harmony of movement | 12 | I like to adapt my movements to match the rhythm of the music. |
| Ideomotor representation and self-control | 13 | I imagine movements before executing them in order to make them appear more aesthetic. |
| Ideomotor representation and self-control | 14 | Mental visualization of the routine helps my aesthetic performance during competitions. |
| Ideomotor representation and self-control | 15 | I feel my movements are more precise when I focus on the aesthetic aspects of my body. |
| Ideomotor representation and self-control | 16 | I believe that improving motor control can make my movements more fluid and elegant. |
| Posture and body line | 17 | I consider that body posture influences my aesthetic performance. |
| Posture and body line | 18 | I attach great importance to my body line during routines. |
| Posture and body line | 19 | I correct my posture when I feel insecure during execution. |
| Posture and body line | 20 | I focus on maintaining a correct body line throughout all movements. |

**EBAS scoring procedure**

The global EBAS score was calculated as the mean of the 20 items. No reverse coding was applied, because all items were formulated in the same direction. Section-level means may be used descriptively to characterize the response profile, but in the present article the global EBAS score was used as the main indicator of perceived body aesthetics.

**S1.2. Movement Imagery Questionnaire-3 (MIQ-3)**

Ideomotor representation was assessed using the Movement Imagery Questionnaire-3 (MIQ-3), following the structure of the original MIQ-3 instrument. The questionnaire was administered in Romanian, with minor wording adaptations for linguistic clarity and age-appropriate comprehension. The instrument includes 12 items and evaluates the ease with which participants generate motor images in three modalities: internal visual imagery, external visual imagery, and kinesthetic imagery.

For each item, the participant first performed the described movement once, then returned to the starting position and completed the corresponding imagery task. Depending on the item, the task required the participant to imagine the movement from an internal visual perspective, imagine the movement from an external visual perspective, or feel the movement without actually performing it.

**Response scale**

Responses were provided on a 7-point scale. For visual imagery tasks, the scale ranged from:

1 = very hard to see

to
7 = very easy to see

For kinesthetic imagery tasks, the scale ranged from:

1 = very hard to feel

to
7 = very easy to feel

Higher scores indicate greater ease in generating the corresponding motor image.

**MIQ-3 dimensions and scoring**

The MIQ-3 provides scores for three imagery modalities. Each dimension is represented by four items. Scores were calculated separately for each dimension as the mean of the corresponding items.

Based on the imagery task described in each item, the dimensional structure is shown in Appendix Table S1.3.

**Appendix Table S1.3.** MIQ-3 dimensions and scoring

| **Dimension** | **Description** | **Items** |
| --- | --- | --- |
| Internal visual imagery | Seeing the movement from a first-person perspective, as if performing and seeing the action through one’s own eyes. | 2, 5, 8, 11 |
| External visual imagery | Seeing the movement from a third-person perspective, as if watching oneself on video. | 3, 6, 9, 12 |
| Kinesthetic imagery | Feeling the movement without actually performing it. | 1, 4, 7, 10 |

Note. Dimension-level scores were calculated as the mean of the four items corresponding to each modality: internal visual imagery = (Item 2 + Item 5 + Item 8 + Item 11) / 4; external visual imagery = (Item 3 + Item 6 + Item 9 + Item 12) / 4; and kinesthetic imagery = (Item 1 + Item 4 + Item 7 + Item 10) / 4.

The global MIQ-3 score used in the pre–post analysis was calculated as the mean of the 12 items.

**Supplementary Appendix S2. Detailed computation procedure for the Coefficient of Harmony (CA) and the Weighted Coefficient of Harmony (CA-P)**

The Coefficient of Harmony (CA) and the Weighted Coefficient of Harmony (CA-P) were calculated as descriptive and exploratory indicators of the internal balance among the three central dimensions analyzed in the study: whole-routine performance, body aesthetics, and ideomotor representation. These indicators were not used as independent outcome scales, but as synthetic descriptors of the degree of reciprocal support among the components of each athlete’s functional profile.

**S2.1. Variables included in the computation**

The variables included in the computation of CA and CA-P are presented in Appendix Table S2.1.

**Appendix Table S2.1.** Variables included in CA and CA-P computation

| **Variable** | **Description** |
| --- | --- |
| Total score / ST | Total whole-routine score, used as the operational indicator of motor control in a competition-specific context |
| EBAS | Global score of the Evaluation of Body Aesthetics in Sports |
| MIQ-3 | Global score of the Movement Imagery Questionnaire-3 |

The same variables were used at T0 and T2.

**S2.2. Standardization procedure**

To allow comparison between T0 and T2 using the same reference base, raw values for Total score, EBAS, and MIQ-3 were transformed into standardized z scores using the parameters of the initial assessment moment (T0):

z = (X − μ_T0) / σ_T0

where X represents the raw value, μ_T0 the mean of the group at T0, and σ_T0 the standard deviation of the group at T0.

The T0 reference parameters used in the computation are presented in Appendix Table S2.2.

**Appendix Table S2.2.** T0 reference parameters used for standardization

| **Variable** | **μ_T0** | **σ_T0** |
| --- | --- | --- |
| Total score | 16.0556 | 1.09643 |
| EBAS | 3.9778 | 0.22928 |
| MIQ-3 | 5.2022 | 0.52538 |

This procedure ensured that both T0 and T2 values were interpreted relative to the same baseline distribution.

**S2.3. Computation of the Coefficient of Harmony (CA)**

For each athlete and each testing moment, the three standardized scores were grouped into an individual profile:

z_ST, z_EBAS, z_MIQ-3

The internal dispersion of this profile was then calculated as the standard deviation of the three z scores:

SD(z_ST, z_EBAS, z_MIQ-3)

The maximum empirical dispersion observed in the combined T0–T2 dataset was used as the normalization reference:

SDmax = max SD(z_ST, z_EBAS, z_MIQ-3) across all T0 and T2 profiles

The Coefficient of Harmony was calculated using the formula:

CA = 1 − [SD(z_ST, z_EBAS, z_MIQ-3) / SDmax]

Higher CA values indicate a more internally balanced profile among whole-routine performance, EBAS, and MIQ-3. Lower CA values indicate greater dispersion among the three components.

**S2.4. Computation of the Weighted Coefficient of Harmony (CA-P)**

The Weighted Coefficient of Harmony (CA-P) was calculated using the same logic as CA, but the three standardized components were weighted according to their relative importance in the analytical model of the study.

The weights used for CA-P computation are presented in Appendix Table S2.3.

**Appendix Table S2.3.** Weights used for CA-P computation

| **Component** | **Weight** |
| --- | --- |
| Total score / ST | 0.26 |
| EBAS | 0.33 |
| MIQ-3 | 0.41 |

Each standardized score was multiplied by its corresponding weight:

z′ = z × p

Thus, the weighted profile for each athlete was:

z′_ST, z′_EBAS, z′_MIQ-3

The internal dispersion of the weighted profile was calculated as:

SDp(z′_ST, z′_EBAS, z′_MIQ-3)

The maximum empirical value of SDp observed in the combined T0–T2 dataset was used as the normalization reference:

SDmax,p = max SDp across all T0 and T2 weighted profiles

In the present computation:

SDmax,p = 0.60

The Weighted Coefficient of Harmony was calculated using the formula:

CA-P = 1 − [SDp(z′_ST, z′_EBAS, z′_MIQ-3) / SDmax,p]

Higher CA-P values indicate a more internally balanced weighted profile, whereas lower values indicate greater dispersion among the weighted components.

**S2.5. Computation trace**

**Appendix Table S2.4.** Computation trace for CA and CA-P

| **Step** | **Operation** | **Output** |
| --- | --- | --- |
| 1 | Selection of raw variables | Total score, EBAS, MIQ-3 |
| 2 | Standardization using T0 parameters | z_ST, z_EBAS, z_MIQ-3 |
| 3 | Calculation of internal dispersion | SD of the three standardized scores |
| 4 | Normalization to maximum empirical dispersion | SD / SDmax |
| 5 | Inversion of the normalized dispersion | CA = 1 − (SD / SDmax) |
| 6 | Application of component weights | z′ = z × p |
| 7 | Calculation of weighted internal dispersion | SDp |
| 8 | Normalization and inversion of weighted dispersion | CA-P = 1 − (SDp / SDmax,p) |

**S2.6. Interpretation**

CA and CA-P were interpreted as descriptive and exploratory indicators of the internal configuration of the athlete’s functional profile. They do not express the absolute level of performance. Instead, they indicate the degree to which the three analyzed dimensions—whole-routine performance, body aesthetics, and ideomotor representation—are internally balanced.

A decrease in CA or CA-P does not automatically indicate regression. It may reflect a temporary increase in dispersion among components, for example when one dimension develops more rapidly than the others. Conversely, a higher value indicates a more balanced relationship among the analyzed components, but not necessarily a higher absolute level of performance.

**Supplementary Results and Statistical Outputs**

The following supplementary results provide detailed statistical and individual-level outputs supporting the findings reported in the main manuscript. These materials include detailed inferential statistics, participant-level pre–post changes, Reliable Change Index analyses, individual CA and CA-P values, and the full correlation matrix. In the main manuscript, the corresponding results are reported in a condensed form in order to reduce duplication between text, tables, and figures.

**Supplementary Table S5.** Individual whole-routine results at T0 and T2

| **Subject** | **Total score T0** | **Total score T2** | **Scripted difficulty (D)** | **D_T0** | **D_T2** | **A+E_T0** | **A+E_T2** |
| --- | --- | --- | --- | --- | --- | --- | --- |
| J4_01 | 16.800 | 17.500 | 5.500 | 3.700 | 5.100 | 13.100 | 12.400 |
| J3_01 | 17.200 | 18.850 | 7.800 | 5.200 | 6.100 | 12.000 | 12.750 |
| J3_02 | 16.050 | 16.700 | 6.900 | 3.400 | 4.200 | 12.650 | 12.500 |
| J3_03 | 13.900 | 18.300 | 5.500 | 2.900 | 3.700 | 11.000 | 14.600 |
| J3_04 | 16.900 | 17.850 | 5.800 | 4.000 | 5.700 | 12.900 | 12.150 |
| J3_05 | 16.900 | 17.500 | 8.700 | 4.300 | 5.500 | 12.600 | 12.000 |
| J3_06 | 15.250 | 15.700 | 6.800 | 5.200 | 4.000 | 10.050 | 11.700 |
| J3_07 | 15.100 | 16.500 | 5.800 | 3.800 | 3.600 | 11.900 | 12.900 |
| J2_01 | 16.400 | 16.450 | 6.900 | 5.900 | 5.100 | 10.500 | 11.350 |

*Note. T0 = initial assessment; T2 = final post-intervention assessment; D = difficulty; A+E = composite execution score obtained by summing the artistic and technical execution components. Total score represents the final score of the whole routine, calculated according to the judging logic applied, including any penalties if applicable. For J3_07, the T0 total score includes cumulative penalties of 0.600 points (3.800 + 11.900 − 0.600 = 15.100), whereas no penalties were recorded at T2 (3.600 + 12.900 = 16.500).*

**Supplementary Table S6.** Detailed inferential statistics for pre–post comparisons

| **Variable** | **Test** | **Comparison** | **Test statistic** | **df** | **p** | **Effect size** |
| --- | --- | --- | --- | --- | --- | --- |
| Global MIQ-3 score | Wilcoxon paired-samples test | T0–T2 | Z = -2.25 | — | 0.024 | r = 0.75 |
| EBAS global score | Paired-samples t-test | T0–T2 | t = -1.43 | 8 | 0.189 | Cohen’s d = -0.48 |
| Difficulty (D) | Paired-samples t-test | T0–T2 | t = -1.51 | 8 | 0.167 | Cohen’s d = -0.51 |
| Composite execution score (A+E) | Paired-samples t-test | T0–T2 | t = -1.33 | 8 | 0.218 | Cohen’s d = -0.45 |

*Note. T0 = initial assessment; T2 = final post-intervention assessment; MIQ-3 = Movement Imagery Questionnaire-3; EBAS = Evaluation of Body Aesthetics in Sports; D = difficulty; A+E = composite execution score obtained by summing the artistic and technical execution components. Effect size for the Wilcoxon paired-samples test was calculated as r = |Z| / √N. Cohen’s d values were interpreted using conventional thresholds.*

**Supplementary Table S7.** Individual evolution of MIQ-3, EBAS, and total score between T0 and T2

| **Subject** | **MIQ-3_T0** | **MIQ-3_T2** | **EBAS_T0** | **EBAS_T2** | **Total score_T0** | **Total score_T2** |
| --- | --- | --- | --- | --- | --- | --- |
| J4_01 | 3.92 | 4.83 | 3.55 | 3.55 | 16.80 | 17.50 |
| J3_01 | 5.33 | 5.75 | 3.90 | 4.50 | 17.20 | 18.85 |
| J3_02 | 5.58 | 6.17 | 4.15 | 4.55 | 16.05 | 16.70 |
| J3_03 | 5.17 | 6.08 | 3.90 | 4.70 | 13.90 | 18.30 |
| J3_04 | 5.58 | 5.75 | 4.15 | 5.00 | 16.90 | 17.85 |
| J3_05 | 5.08 | 5.25 | 3.85 | 4.40 | 16.90 | 17.50 |
| J3_06 | 5.58 | 5.33 | 4.15 | 3.60 | 15.25 | 15.70 |
| J3_07 | 5.08 | 5.42 | 3.85 | 3.50 | 15.10 | 16.50 |
| J2_01 | 5.50 | 5.75 | 4.30 | 4.20 | 16.40 | 16.45 |

*Note. T0 = initial assessment; T2 = final post-intervention assessment; MIQ-3 = Movement Imagery Questionnaire-3; EBAS = Evaluation of Body Aesthetics in Sports; Total score = final whole-routine score.*

**Supplementary Table S8.** Individual progress analysis for MIQ-3 using the Reliable Change Index (RCI)

| **Subject** | **MIQ-3_T0** | **MIQ-3_T2** | **ΔMIQ-3** | **RCI**  **(r = 0.844)** | **Significance** |
| --- | --- | --- | --- | --- | --- |
| J4_01 | 3.92 | 4.83 | 0.91 | 3.10 | Significant |
| J3_01 | 5.33 | 5.75 | 0.42 | 1.43 | Not significant |
| J3_02 | 5.58 | 6.17 | 0.59 | 2.01 | Significant |
| J3_03 | 5.17 | 6.08 | 0.91 | 3.10 | Significant |
| J3_04 | 5.58 | 5.75 | 0.17 | 0.58 | Not significant |
| J3_05 | 5.08 | 5.25 | 0.17 | 0.58 | Not significant |
| J3_06 | 5.58 | 5.33 | -0.25 | -0.85 | Not significant |
| J3_07 | 5.08 | 5.42 | 0.34 | 1.16 | Not significant |
| J2_01 | 5.50 | 5.75 | 0.25 | 0.85 | Not significant |

*Parameters used: SD_T0 = 0.525; SE_diff = SD_T0 × √(2 × (1 − r)) = 0.293.*
*Note. T0 = initial assessment; T2 = final post-intervention assessment; MIQ-3 = Movement Imagery Questionnaire-3; RCI = Reliable Change Index.*

**Supplementary Table S9.** Individual progress analysis for EBAS using the Reliable Change Index (RCI)

| **Subject** | **EBAS_T0** | **EBAS_T2** | **ΔEBAS** | **RCI (α_T0 = 0.717)** | **Significance** |
| --- | --- | --- | --- | --- | --- |
| J4_01 | 3.55 | 3.55 | 0.00 | 0.00 | Not significant |
| J3_01 | 3.90 | 4.50 | 0.60 | 3.46 | Significant |
| J3_02 | 4.15 | 4.55 | 0.40 | 2.31 | Significant |
| J3_03 | 3.90 | 4.70 | 0.80 | 4.61 | Significant |
| J3_04 | 4.15 | 5.00 | 0.85 | 4.90 | Significant |
| J3_05 | 3.85 | 4.40 | 0.55 | 3.17 | Significant |
| J3_06 | 4.15 | 3.60 | -0.55 | -3.17 | Significant (regression) |
| J3_07 | 3.85 | 3.50 | -0.35 | -2.02 | Significant (regression) |
| J2_01 | 4.30 | 4.20 | -0.10 | -0.58 | Not significant |

Note. r = α (Cronbach’s alpha) determined at T0. The EBAS_T0 and EBAS_T2 scores used in the RCI calculation represent the global EBAS score (EBAS-20), calculated as the mean of the 20 instrument items. T0 = initial assessment; T2 = final post-intervention assessment; EBAS = Evaluation of Body Aesthetics in Sports; RCI = Reliable Change Index.

**Supplementary Table S10. Individual CA and CA-P values at T0 and T2**

| **Subject** | **CA_T0** | **CA_T2** | **ΔCA** | **CA-P_T0** | **CA-P_T2** | **ΔCA-P** |
| --- | --- | --- | --- | --- | --- | --- |
| J4_01 | 0.090 | 0.117 | +0.027 | 0.005 | 0.192 | +0.187 |
| J3_01 | 0.619 | 0.560 | -0.059 | 0.682 | 0.722 | +0.040 |
| J3_02 | 0.766 | 0.469 | -0.297 | 0.736 | 0.388 | -0.348 |
| J3_03 | 0.436 | 0.579 | +0.143 | 0.570 | 0.569 | -0.001 |
| J3_04 | 0.986 | 0.000 | -0.986 | 0.922 | 0.000 | -0.922 |
| J3_05 | 0.621 | 0.508 | -0.113 | 0.666 | 0.527 | -0.139 |
| J3_06 | 0.535 | 0.468 | -0.067 | 0.556 | 0.451 | -0.105 |
| J3_07 | 0.825 | 0.211 | -0.614 | 0.889 | 0.208 | -0.681 |
| J2_01 | 0.687 | 0.795 | +0.108 | 0.681 | 0.717 | +0.036 |

Note. T0 = initial assessment; T2 = final post-intervention assessment; CA = Coefficient of Harmony; CA-P = Weighted Coefficient of Harmony; Δ = T2–T0 difference. CA and CA-P were calculated using Total score, EBAS, and MIQ-3.

**Supplementary Table S11.** Correlations between CA, CA-P, and competitive performance

| **Variable** | **Statistic** | **CA post** | **CA-P post** | **NC score** | **NC rank** | **Best secondary score** |
| --- | --- | --- | --- | --- | --- | --- |
| CA post | Correlation coefficient | 1.000 | 0.717* | 0.250 | 0.119 | 0.017 |
|  | p | — | 0.030 | 0.516 | 0.761 | 0.966 |
|  | N | 9 | 9 | 9 | 9 | 9 |
| CA-P post | Correlation coefficient | 0.717* | 1.000 | 0.117 | 0.424 | 0.100 |
|  | p | 0.030 | — | 0.765 | 0.256 | 0.798 |
|  | N | 9 | 9 | 9 | 9 | 9 |
| NC score | Correlation coefficient | 0.250 | 0.117 | 1.000 | -0.576 | 0.633 |
|  | p | 0.516 | 0.765 | — | 0.104 | 0.067 |
|  | N | 9 | 9 | 9 | 9 | 9 |
| NC rank | Correlation coefficient | 0.119 | 0.424 | -0.576 | 1.000 | -0.254 |
|  | p | 0.761 | 0.256 | 0.104 | — | 0.509 |
|  | N | 9 | 9 | 9 | 9 | 9 |
| Best secondary score | Correlation coefficient | 0.017 | 0.100 | 0.633 | -0.254 | 1.000 |
|  | p | 0.966 | 0.798 | 0.067 | 0.509 | — |
|  | N | 9 | 9 | 9 | 9 | 9 |

Note. Values are Spearman correlation coefficients (ρ). The level of statistical significance was set at p < 0.05, two-tailed. CA post = Coefficient of Harmony at T2; CA-P post = Weighted Coefficient of Harmony at T2; NC score = score obtained at the National Championship; NC rank = ranking position at the National Championship; Best secondary score = best score obtained in the secondary competitions of the 2023 season, excluding the National Championship. *p < 0.05.
